# Synthesis and antiproliferative activity of 2-oxo-3-phenylquinoxaline derivatives and related compounds against colon cancer†

**DOI:** 10.1039/d4ra06822j

**Published:** 2024-11-08

**Authors:** M. S. Gomaa, Abdulghany H. A. Ahmed, S. M. El Rayes, Ibrahim A. I. Ali, Walid Fathalla, Mansour S. Alturki, Abdulaziz Hassan Al Khzem, Atiah H. Almalki, Mohammed F. Aldawsari, F. H. Pottoo, Firdos A. Khan, Mohd Amir

**Affiliations:** a Department of Pharmaceutical Chemistry, College of Clinical Pharmacy, Imam Abdulrahman Bin Faisal University P. O. Box 1982 Dammam 31441 Eastern Province Kingdom of Saudi Arabia; b Chemistry Department, Faculty of Medicinal Science, University of Science and Technology Aden 15201 Yemen; c Department of Chemistry, Faculty of Science, Suez Canal University Ismailia Egypt samir_elrayes@science.suez.edu.eg; d Department of Physical Sciences, Faculty of Engineering, Suez Canal University Ismailia Egypt; e Department of Pharmaceutical Chemistry, College of Pharmacy, Taif University P. O. Box 11099 Taif 21944 Saudi Arabia; f Addiction and Neuroscience Research Unit, College of Pharmacy, Taif University Al-Hawiah Taif 21944 Saudi Arabia; g Department of Pharmaceutics, College of Pharmacy, Prince Sattam Bin Abdulaziz University Al-kharj 11942 Saudi Arabia; h Department of Pharmacology, College of Clinical Pharmacy, Imam Abdulrahman Bin Faisal University, Eastern Province P. O. Box 1982 Dammam 31441 Saudi Arabia; i Department of Stem Cell Biology, Institute for Research & Medical Consultations, (IRMC), Imam Abdul Rahman Bin Faisal University Dammam 31441 Saudi Arabia; j Department of Natural Products, College of Clinical Pharmacy, Imam Abdulrahman Bin Faisal University Dammam 1982 Saudi Arabia

## Abstract

We have designed 17 new 2-oxo-3-phenylquinoxalines *via* the chemoselective Michael reaction of 3-phenylquinoxalin-2(1*H*)-one with acrylic acid derivatives. The ester, ethyl 3-(2-oxo-3-phenylquinoxalin-1(2*H*)-yl)propanoate, was reacted with hydroxylamine and hydrazine to produce *N*-hydroxy-3-(2-oxo-3-phenylquinoxalin-1(2*H*)-yl)propanamide and hydrazide, respectively. Further modifications were made through reactions with isothiocyanates and azide coupling with amines, yielding thiosemicarbazides and *N*-alkyl derivatives. Molecular docking studies identified compound 7j as the most potent binder, fitting well into the active site, with the phenyl ring occupying the S1 pocket and the amino acid chain positioned in the S2 pocket. The synthesized compounds (2a, 4, 7a, 7g, 7d, 7h, 7e, 7b, 7c, 7f, and 7j) were evaluated for their anti-cancer activity on colorectal cancer (HCT-116) cells. Compounds 2a and 7j showed significant reductions in cell viability, with IC50 values of 28.85 ± 3.26 μg mL^−1^ and 26.75 ± 3.50 μg mL^−1^, respectively. Image analysis of HCT-116 cells treated with 60 μg mL^−1^ of compound 7j for 48 hours revealed notable morphological changes in both nuclei and cells. The number of cells reduced from 447 in the control to 238 in the treated group, with a corresponding reduction in the area covered by cells from 41.9% to 17.6%. Nuclear disintegration and chromatin fragmentation were observed, confirming apoptosis. These results highlight the potent cytotoxic effect of compound 7j.

## Abstract 2

The impact of compound 7j on colon cancer cells (HCT-116). Fig. (A) shows the control cells Fig. (B) shows the treatment cells. The arrows show chromatin condensation, nuclear augmentation, and formation of apoptotic bodies.

## Graphical abstract

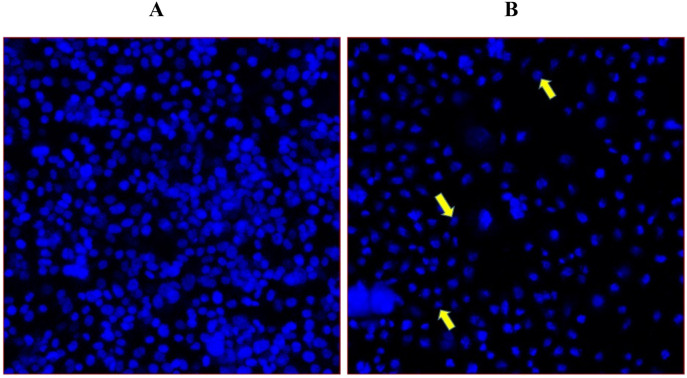

## Introduction

Colorectal cancer (CRC) ranks as the second most significant contributor to global cancer-related deaths. According to the Global Cancer Statistics 2020, it is reported that this particular type of cancer is responsible for 9.2% of all cancer-related fatalities.^1^ The regulation of carcinogenesis involves the careful control of antioxidant enzymes and matrix-degrading enzymes, specifically matrix metalloproteinases (MMPs). The requirement for tumor invasion and metastasis is the degradation of extracellular matrix (ECM) proteins, such as collagen, proteoglycan, laminin, elastin, and fibronectin. MMPs possess the capability to degrade a wide range of ECM components. Additionally, many MMPs play a significant role in processes like angiogenesis, differentiation, proliferation, and death. Therefore, MMPs have a crucial role in regulating tumor growth, both locally and in distant metastases. Consequently, these enzymes are regarded as significant targets for cancer treatment.^2^

Inhibitors of MMPs (MMPI) were investigated across various cancer types. Nevertheless, despite the compelling preclinical evidence, all clinical trials proved unsuccessful as a result of both insufficient effectiveness and the occurrence of significant adverse reactions.^3–5^ One crucial factor in elucidating the failure lies in the potential anticancer effects exhibited by some MMPs. However, it is plausible that the utilization of broad-spectrum MMPIs during the early trials could impede certain MMPs, thereby leading to tumor progression.^6^ In light of the expanding understanding of matrix metalloproteinases (MMPs) in the processes of tumor invasion and metastasis, as well as their larger involvement in cancer biology, there is presently a focus on the development of narrow-spectrum MMP inhibitors (MMPIs) that offer enhanced safety and selectivity.^7^

MMPs are known to have a crucial function in both the onset and progression of colorectal cancer (CRC). Prominent MMPs, namely MMP-1, MMP-2, MMP-7, MMP-8, MMP-9, MMP-12, MMP-13, MMP-14, and MMP-21, have been identified in patients with CRC. The production of these MMPs is often associated with a worse prognosis. Moreover, the genes and proteins associated with MMPs are known to play a significant part in the progression from pre-cancerous lesions and polyps to advanced CRC through intricate pathways. Consequently, the utilization of MMPIs could potentially serve as a crucial approach in the treatment and prevention of CRC.^8^

The correlation between the overexpression of MMP-2 and upregulation of MMP-9 and the development and prognosis of CRC has been well studied in the literature and proven MMP-2 and MMP-9 to play a pivotal role in CRC development and metastasis.^9–12^ The initial inhibitors consisted of either small-molecule analogs of the naturally occurring ligands of MMPs or their peptidomimetics that were connected to zinc-binding groups (ZBGs), such as hydroxamic acid, in order to deactivate the enzyme.^13^ Nevertheless, the aforementioned compounds did not advance successfully in clinical trials due to their lack of selectivity, resulting in toxicity,^14^ as well as their inadequate pharmacokinetic features.^15^ Consequently, other inhibitors lacking hydroxamate groups have been introduced, including those containing carboxylates, hydrazides, thiols, and phosphorus-based ZBGs.^16^ In addition, the researchers successfully produced inhibitors that do not bind to zinc, yet nevertheless demonstrate the capability to bind to the active site of MMP.^17^ These inhibitors exhibit common structural characteristics, characterized by the presence of planar rings that incorporate amino and carbonyl groups, facilitating hydrophilic interactions.^18^

**Fig. 1:**
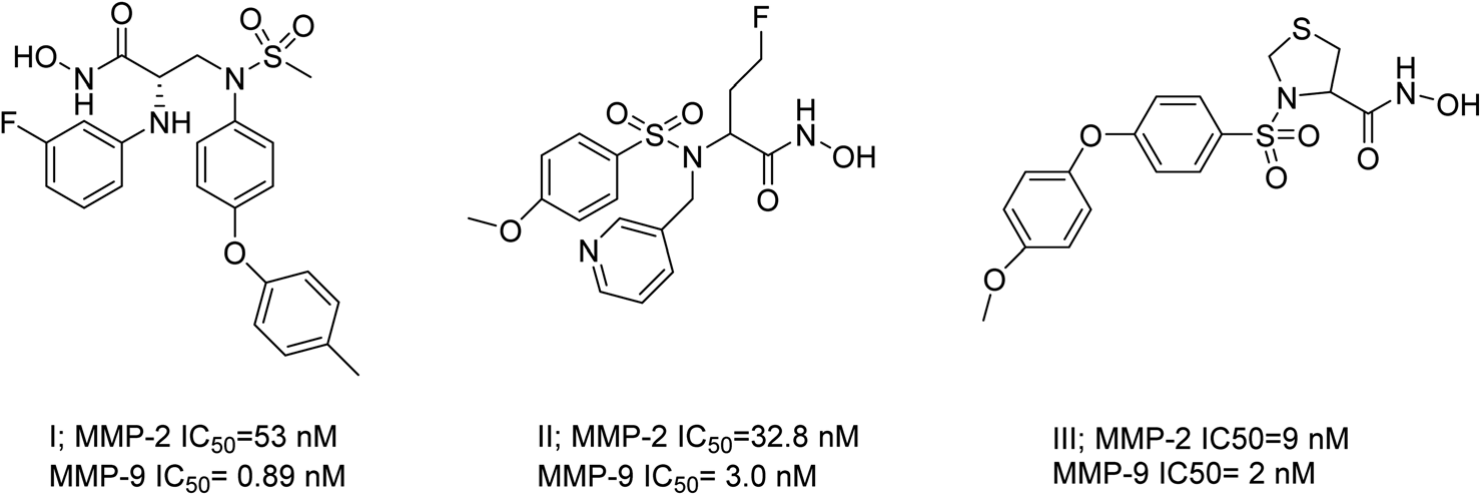
Selective dual MMP-2 and MMP-9 inhibitors.^19^

**Fig. 2:**
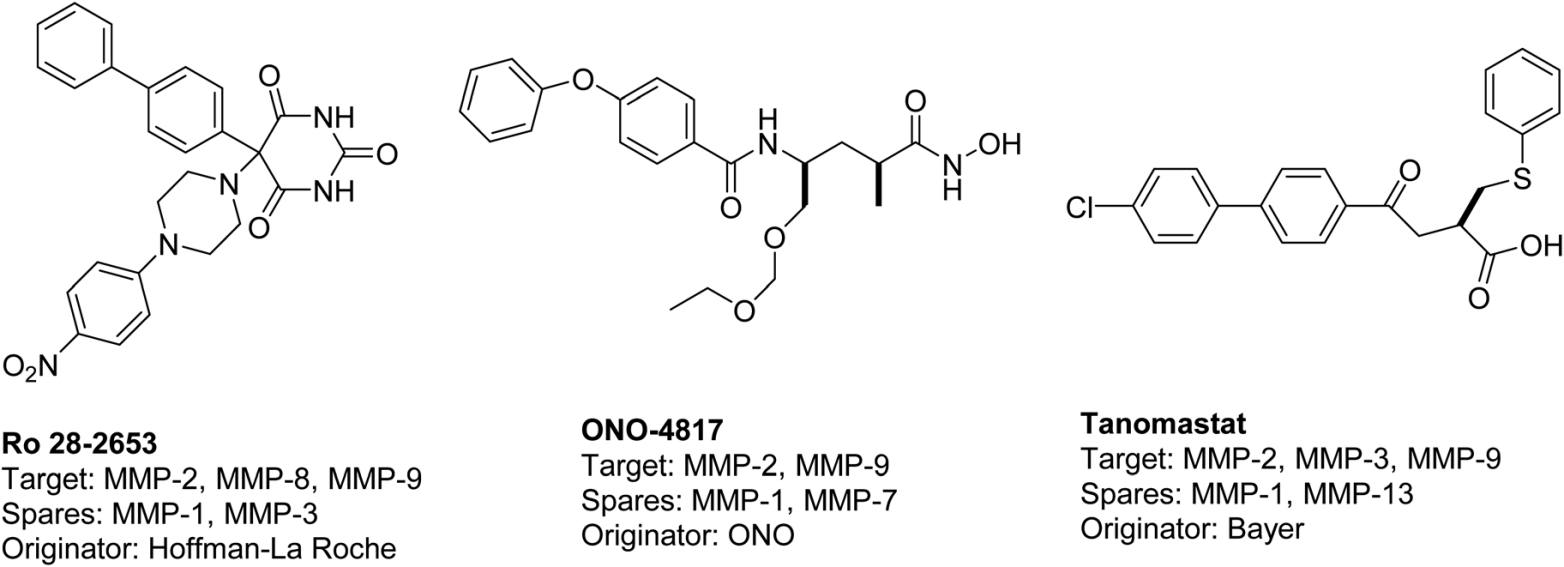
Selective dual MMP-2 and MMP-9 inhibitors progressed in clinical trials.^20^

The existing literature predominantly reports that hydroxamic acid groups, which function as the Zn binding moiety, are commonly observed in highly effective inhibitors of MMP-2 and MMP-9. Nevertheless, there is a lack of available data regarding the precise selectivity of these drugs against MMPs or the current status of these compounds in clinical studies^19^ (Fig. 1). Various pharmaceutical companies have successfully produced a range of MMP-2 and MMP-9 inhibitors, which are primarily non-hydroxamic acid derivatives. However, it is important to note that none of these inhibitors have received approval from the FDA as of yet^20^ (Fig. 2).

**Fig. 3:**
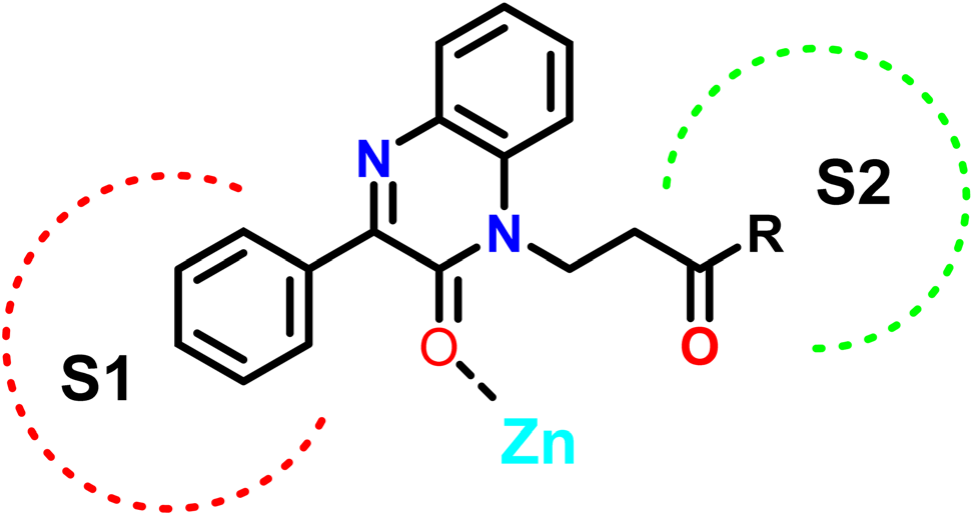
Design of the quinoxalinone compounds.

In this study, we employed a ligand-based strategy to develop our compounds. This involved analyzing the structures of previously reported selective inhibitors of the targeted MMPs and incorporating key substructures onto the 2-quinoxaline-one scaffold. Hydroxamates were deliberately omitted as a zinc binding group in order to prevent the inhibition of a wide range of MMPs and to impart selectivity to the compounds being designed. The Zn binding group in our compounds is represented by the modified 2-quinoxaline-one scaffold, while the strategically selected substituents are intended to selectively occupy the pockets S1′ and S2′ of the enzymes, as depicted in Fig. 3.

The 2-quinoxaline-one ring represents the core structure that binds the Zn with its carbonyl oxygen. The compound has its phenyl proximal enough to the zinc to change the water entity and strengthen the compound binding to the zinc atom. The substitution on the quinazoline nitrogen is manipulated to include a hydrophilic arm that ends with various substituents that will serve as a specificity loop to control the compounds' subpocket occupation leading to selectivity against other MMPs. In the current study, we synthesized and investigated the influence of a series of 2-oxo-3-phenylquinoxaline derivatives on the colorectal cancer (HCT-116) cells to investigate their potential application in colorectal cancer treatment.

Quinoxalines possess a wide array of pharmaceutical activities such as anticancer, antimicrobial, and anti-inflammatory.^21–27^ Our research group in their diligent search for the synthesis of compounds with significant properties focused on chemo selective reactions of heterocyclic amides for drug development.^28–33^ 3-Phenylquinoxalin-2(1*H*)-one has proven to be a versatile synthon for the formation of highly biologically active compounds. The reaction of 3-phenylquinoxalin-2(1*H*)-one with electrophiles; methyl iodide, allyl bromide, propargyl bromide, phenacyl bromide, chloroacetanides, pentyl bromide, ethyl chloroacetate, ethyl bromopropanoate and ethyl bromobutanoate in the presence of a base; potassium carbonate, sodium hydride, cesium carbonate and sodium ethanolate in DMF, acetonitrile, ethanol and acetone always afforded the *N*-alkyl substituted quinoxaline.^34–42^ Only, few cases were reported to afford a mixture of both *N*-and *O*-alkyl substituted quinoxaline; the reaction of 3-phenylquinoxalin-2(1*H*)-one with carbomyl chloride in pyridine under reflux condition and with bromo sugar in the presence of sodium hydride in DMF under reflux condition.^43,44^ Other reactions concerning 3-phenylquinoxalin-2(1*H*)-one involve cleavage of the oxygen atom *via* chlorination or thiation and saturation of the fused piperazine ring.^45–47^ As far as, our knowledge no reaction of 3-phenylquinoxalin-2(1*H*)-one with methyl acrylate, acrylonitrile or activated double bond under Michael reaction condition were reported to date. In an attempt to find more promising quinoxaline derivatives for biological evaluation we now report the preparation of *N*-hydroxy-3-(2-oxo-3-phenylquinoxalin-1(2*H*)-yl)propanamide, *N*-3-(2-oxo-3-phenylquinoxalin-1(2*H*)-yl) propanhydrazide, thiosemicarbazides and *N*-alkyl-3-(2-oxo-3-phenylquinoxalin-1(2*H*)-yl)propanamides *via* Michael reaction of acrylic acid with 3-phenylquinoxalin-2(1*H*)-one.

## Results and discussion

**Scheme 1:**
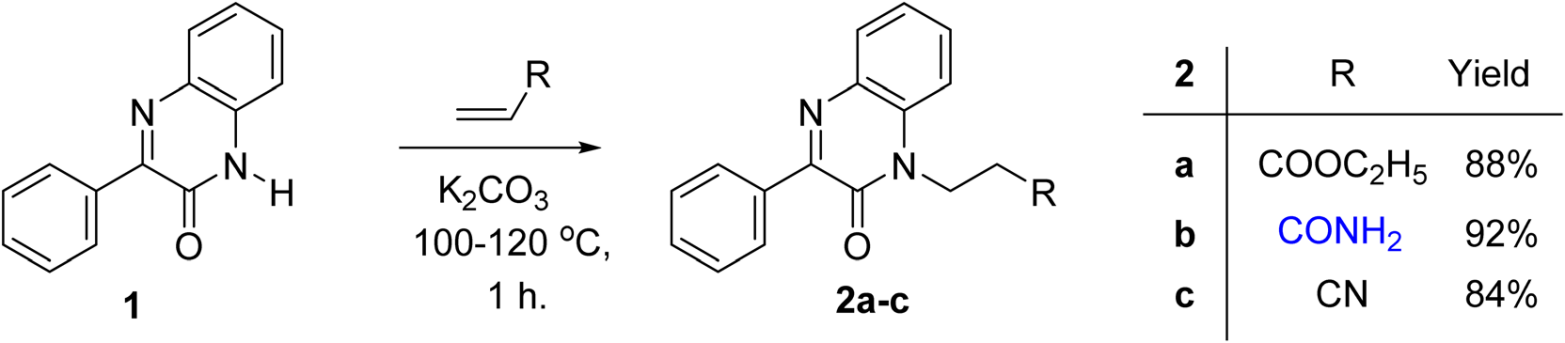
Preparation of 3-(2-oxo-3-phenylquinoxalin-1(2*H*)-yl)propanoic acid derivatives 2a–c.

The reaction of acrylic acid derivatives; ethyl acrylate, acrylamide and acrylonitrile termed as hard electrophiles with 3-phenylquinoxalin-2(1*H*)-one (1) in the presence of potassium carbonate always gave 3-(2-oxo-3-phenylquinoxalin-1(2*H*)-yl)propanoic acid derivatives 2a–c; ester, amide, and nitrile, respectively in 84–92% yield (Scheme 1). This result and the previously mentioned reactivity of 1 as an ambident nucleophile aggregate the soft and hard nucleophilic character on the nitrogen atom. Also, we should note that this reaction was the first reported Michael reaction to this structure using fusion.

**Scheme 2:**
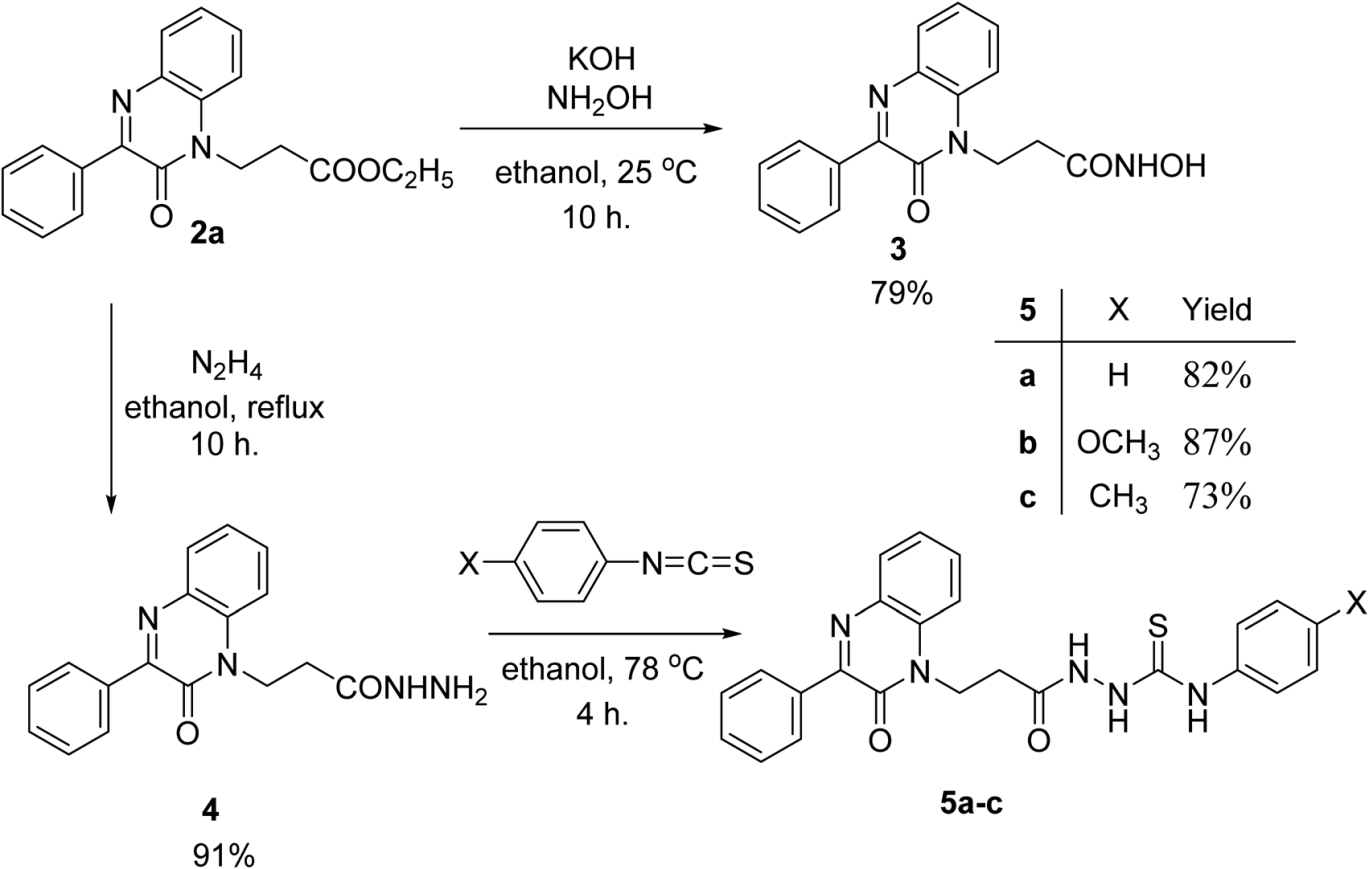
Preparation of *N*-hydroxy-3-(2-oxo-3-phenylquinoxalin-1(2*H*)-yl)propanamide (3) and aryl-1-(3-(2-oxo-3-phenylquinoxalin-1(2*H*)-yl)propanoyl) thiosemicarbazides 5a–c.

Ethyl 3-(2-oxo-3-phenylquinoxalin-1(2*H*)-yl)propanoate (2a) reacted with hydroxyl amine in the presence of potassium hydroxide in ethanol at room temperature to afford the biologically interesting *N*-hydroxy-3-(2-oxo-3-phenylquinoxalin-1(2*H*)-yl)propanamide (3) in 79% yield, Scheme 2. Also, the ester 2a reacted with hydrazine in ethanol under reflux conditions to give 3-(2-oxo-3-phenylquinoxalin-1(2*H*)-yl)propanehydrazide (4), Scheme 2. The hydrazide 4 is an interesting precursor for the structure modification of quinoxaline ring. Thus hydrazide 4 reacted with aryl isothiocyanates; phenyl, 4-methoxyphenyl and *p*-tolyl isothiocyanates to afford 4-aryl-1-(3-(2-oxo-3-phenylquinoxalin-1(2*H*)-yl)propanoyl) thiosemicarbazides 5a–c in excellent yields (Scheme 2).

Azide coupling method could be used to change the structure of the hydrazide 4 by the reaction with nucleophiles. The azide coupling is a reliable method in peptide synthesis and is used as one important tool in the attachment of amines and amino acids *via* peptide bonds. This method has the advantage of reducing the degree of racemization, easily removable by-products, and is applied at low temperatures.^48^

**Scheme 3:**
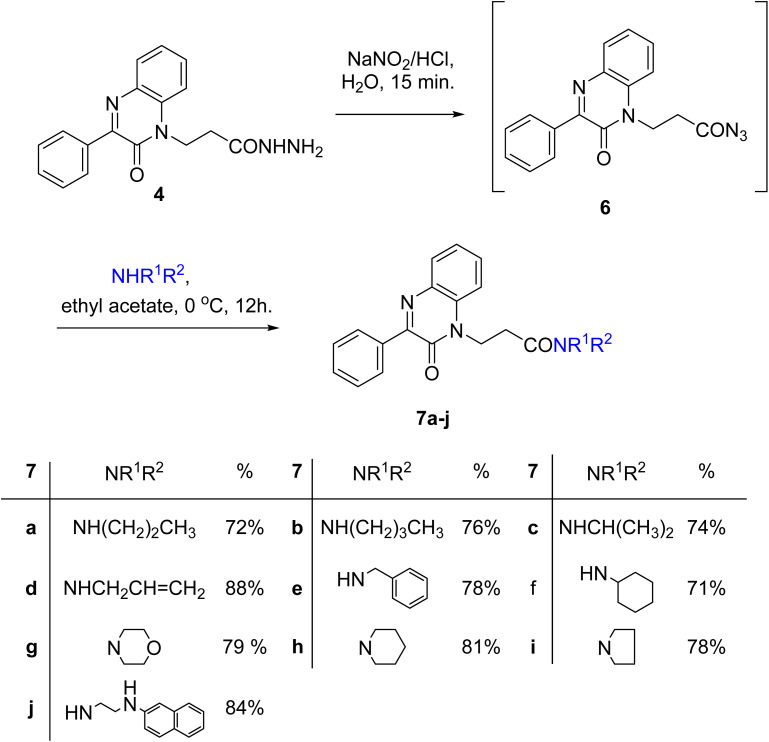
Preparation of *N*-alkyl-3-(2-oxo-3-phenylquinoxalin-1(2*H*)-yl)propanamides 7a–j.

A slurry reaction mixture was produced by adding hydrochloric acid to hydrazide 4 in an ice bath. To this solution cold sodium nitrite solution was added portion wise and stirring was continued for 1 h at 0 °C, and then extracted with ethyl acetate to finally give the *in situ* generated azide 6. The ethyl acetate solution of azide 6 was added to ethyl acetate solution of amines; propyl amine, butyl amine, isopropyl amine, allyl amine, benzylamine, cyclohexyl amine, morpholine, piperidine, pyrrolidine and β-naphthylethylenediamine to finally give *N*-alkyl-3-(2-oxo-3-phenylquinoxalin-1(2*H*)-yl)propanamides 7a–j in moderate to excellent yields (Scheme 3).

The structure assignment of 3-(2-oxo-3-phenylquinoxalin-1(2*H*)-yl)propanoic acid derivatives 2a–c, *N*-hydroxy-3-(2-oxo-3-phenylquinoxalin-1(2*H*)-yl)propanamide (3) and aryl-1-(3-(2-oxo-3-phenylquinoxalin-1(2*H*)-yl)propanoyl) thiosemicarbazides 5a–c and *N*-alkyl-3-(2-oxo-3-phenylquinoxalin-1(2*H*)-yl)propanamides 7a–j was based on ^1^H, ^13^C NMR as well as physicochemical analysis. Thus, ^1^H NMR spectrum of *N*– *N*-isopropyl-3-(2-oxo-3-phenylquinoxalin-1(2*H*)-yl)propanamide (7c) gave two multiplet signals at *δ* 2.66–2.71 and 4.63–4.68 ppm corresponding to CH_2_CO and NCH_2_, respectively, which gave good evidence of the site of alkylation (*N*-substitution). The ^1^H NMR spectrum also shows signals at *δ* 1.11, 4.02–4.13 and 5.70 ppm corresponding to 2CH_3_, CH and NH groups, respectively of the isopropyl amine residue. The ^13^C NMR spectrum of 7c showed signals at *δ* 34.4, 39.6, 41.8, 154.6 and 169.0 ppm corresponding to CH_2_CO, NCH_2_, NCH and two C=O groups, respectively. This gave good evidence for the site of alkylation (propanoate residue) and the introduction of isopropyl amine residue.

### Molecular modeling

**Fig. 4:**
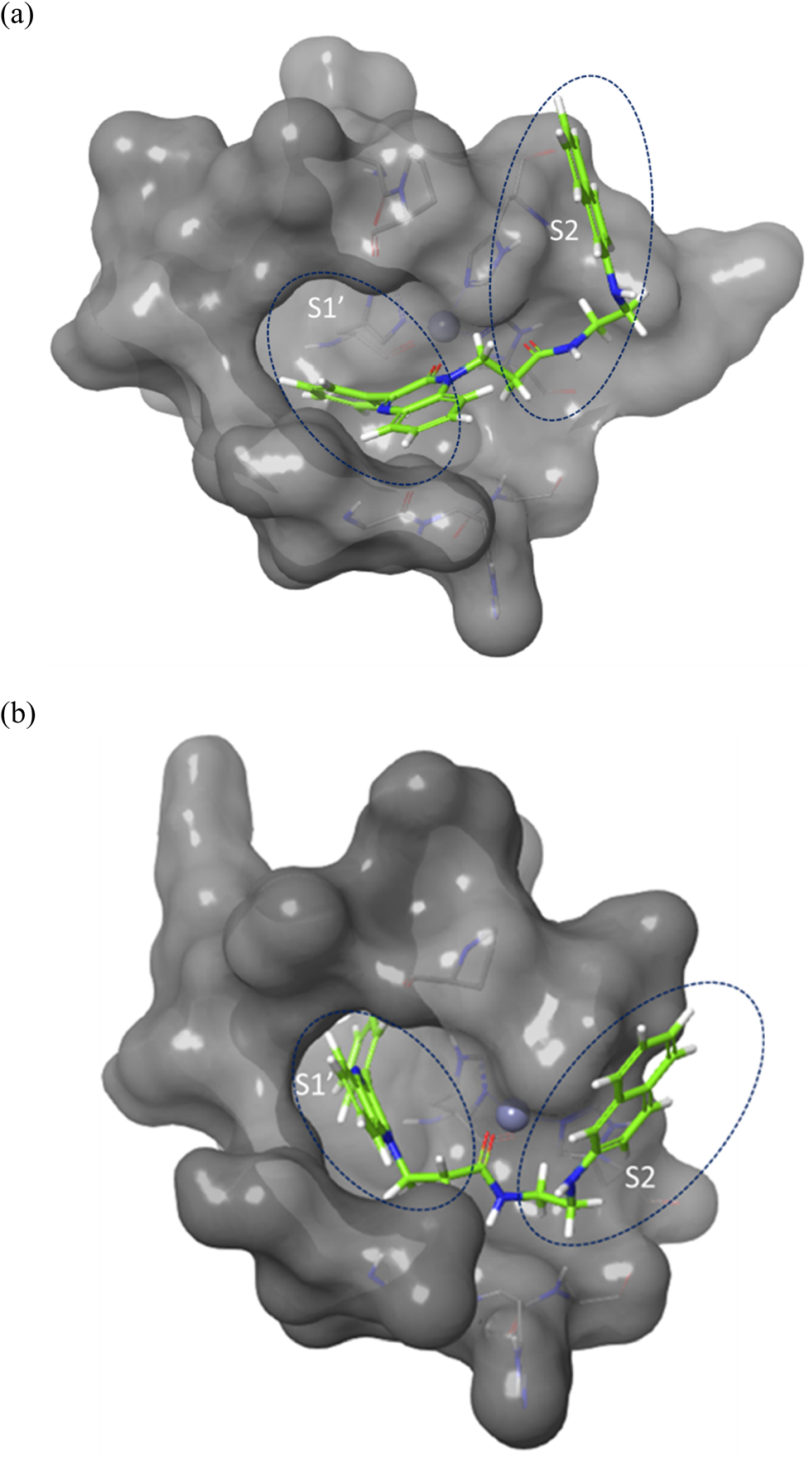
Surface representation of the overlay of 7j in (a) MMP-2 active site (PDB ID: 8H78) and (b) MMP-9 active site (PDB ID: 4H3X). Enzymes sub-pockets are labeled with dashed circles and Zn cation is represented in grey.

Molecular docking was performed to investigate the binding characters of these analogues with MMP-2 and MMP-9 active sites and their correlation with their antiproliferative activity on colon cancer cell lines. To confirm the accuracy of the docking technique, the ligands co-crystallized with MMP-2 and MMP-9 were redocked in their respective active sites. The same docking protocol used to screen the quinoxaline ligands was also used. The expected structures with the least energy for each target were aligned with their corresponding crystalline ligands using Maestro's structure superposition tool. The RMSD for the anticipated binding postures was subsequently computed. The results showed a strong agreement in binding modes, with RMSD values of 2.8 Å for MMP-2 (195 atoms) and 1.9 Å for MMP-9 (69 atoms) (Fig. 4). This initial discovery confirmed the accuracy of Glide in predicting the binding to the active sites of MMP-2 and MMP-9. The docking results were impressive in ranking the most experimentally active compound 7j, as the best binder for both enzymes with an XP score of −7.5 for both targets compared to a score of −13.0, and −7.8 for the MMP-2 and MMP-9 crystal ligands respectively. Compounds 2a, the second experimentally most active compound, showed lower alignment with its XP score where it ranked third and fourth and with docking score of −6.5, and −4.5 in case of MMP-2 and MMP-9 respectively. This correlation was not well established for other compounds based only on their docking scores.

Compound 7j, apart from exhibiting the highest XP score among its analogues, showed an interesting docking pose that followed its initial design. The compound had its phenyl quinoxaline residing in the S1 pocket and the flexible amino acid chain extending in the S2 pocket. The Zn cation was chelated with the quinoxaline oxygen in case of MMP-2 and the side chain carbonyl oxygen in case of MMP-9.

The binding of 7j with the MMP-2 active site showed that the compound is anchored in the active site by binding the Zn cation with its quinoxaline oxygen as well as with forming a π–π stacking with its phenyl ring and His 121. This phenyl ring would also provide a hydrophobic environment that strengthens the bond of the quinoxaline ring with the Zn cation.

**Fig. 5:**
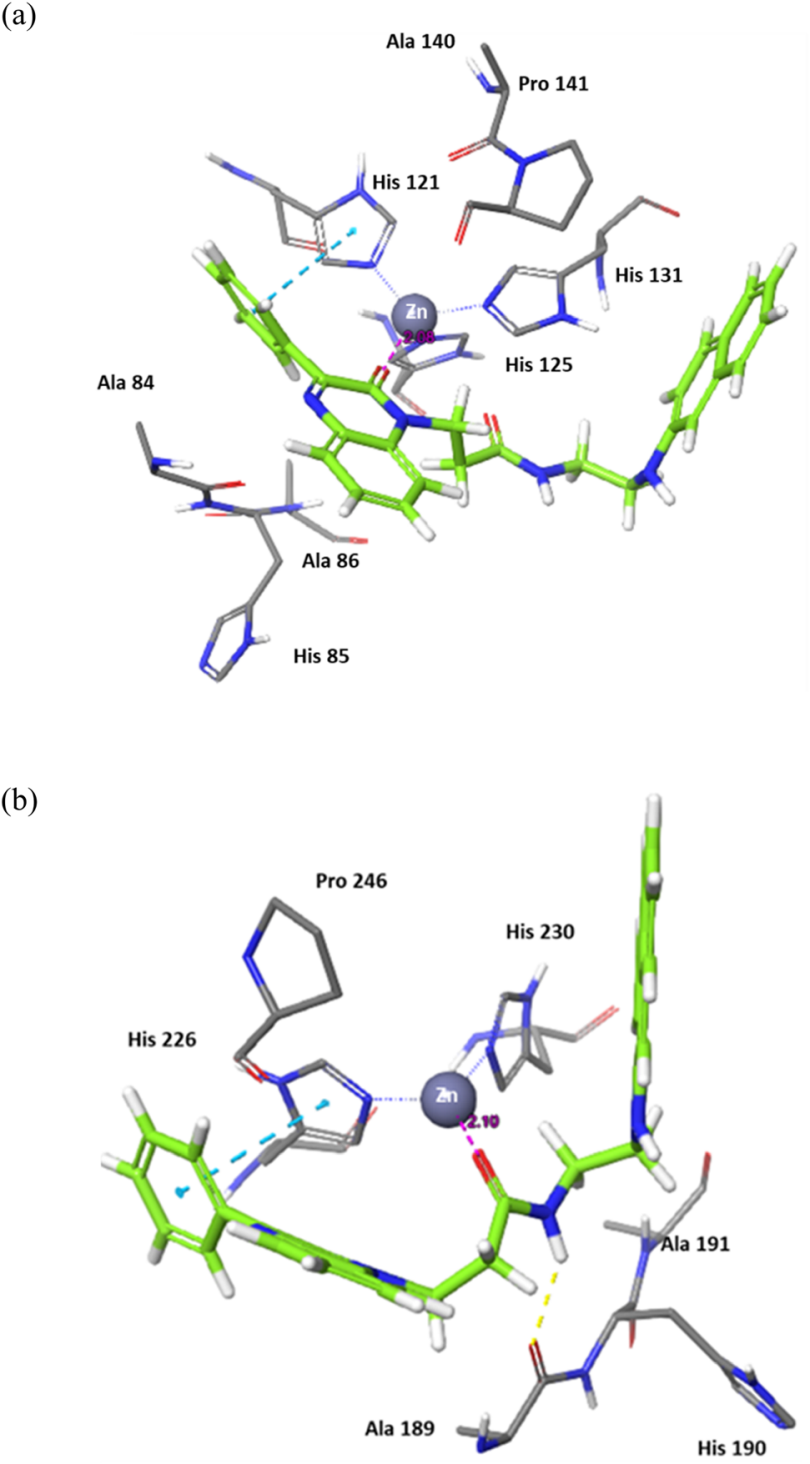
Binding interactions of 7j with (a) MMP-2 active site (PDB ID: 8H78), and (b) MMP-9 active site (PDB ID: 4H3X). Hydrogen bonds are represented as yellow dotted lines, π–π stacking is represented as blue dotted lines, and distances for potential electrostatic attractions are represented as purple dotted lines.

In MMP-9 active site, compound 7j was able to dig further in the S1′ pocket with its phenyl ring and to establish a π–π stacking with its phenyl ring and His 226. In this case, the compound was able to bind the Zn cation with its amino acid side chain oxygen. A hydrogen bond was also noted for the side chain nitrogen and Ala 189 (Fig. 5).

In conclusion, the docking study established key structural features for the quinoxaline compounds. The phenyl group attached to the quinoxaline ring is crucial for its activity as it can engage in π–π stacking interactions with a key histidine residue in both targets. The quinoxaline scaffold plays a crucial role in anchoring the compounds in the active site by potentially binding or attracting the Zn cation due to its electron density. To fit and interact with the S2 pocket and simultaneously bind with the Zn cation, the side chain must be flexible and contain hydrogen donors and acceptors. Further optimization would involve customizing the side chain to enhance the interaction while preserving the overall binding affinity.

### Anti-cancer *in vitro* analysis

**Fig. 6:**
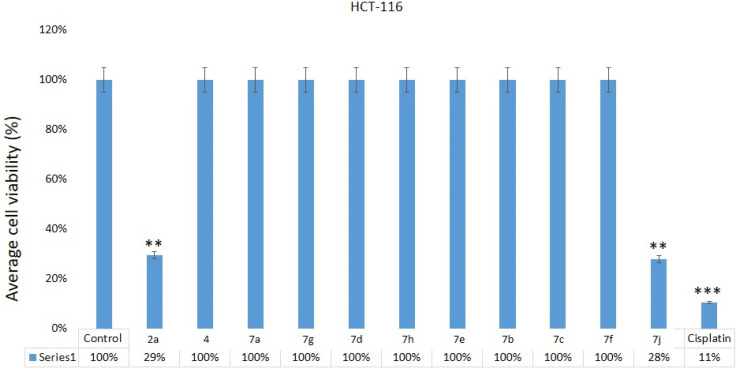
Cell viability assay: MTT assay results showing the influence of treatment of quinoxalines 2a, 4, 7a, 7g, 7d, 7h, 7e, 7b, 7c, 7f, 7j on HCT-116, cells after 48 h of treatment. **p < 0.01. The inhibitory concentration (IC50) for compounds 2a and 7j was 28.85 + 3.26 μg mL^−1^ and 26.75 + 3.50 μg mL^−1^ (Table 1). Whereas IC_50_ for the cisplatin was 10.25 + 1.25 μg mL^−1^ (HCT-116) (Table 1).

The impact of 2a, 4, 7a, 7g, 7d, 7h, 7e, 7b, 7c, 7f, 7j on HCT-116 cells was examined. Post 48 hours of treatment, we found a significant decrease in cancer cells post-treatments of quinoxalines 2a, 7j (Fig. 6) on HCT-116, whereas we did not observe any inhibitory action on HCT-116 cells after treatment of 2a, 4, 7a, 7g, 7d, 7h, 7e, 7b, 7c, 7f, 7j samples. Our results demonstrate that the 2a and 7j showed strong cytotoxicity against HCT-116 cells. There are many studies have done which showed that different nanomaterials and biomaterials produce anti-cancer activities (Table 1).^49^

**Table 1:** IC50 values for HCT-116 cells

Code no.	HCT-116 cells (IC50 μg mL^−1^)
2a	28.85 ± 3.26
4	NI
7a	NI
7g	NI
7d	NI
7h	NI
7e	NI
7b	NI
7c	NI
7f	NI
7j	26.75 ± 3.50
Cisplatin	10.25 ± 1.25

### Anti-apoptotic impact of compounds

**Fig. 7:**
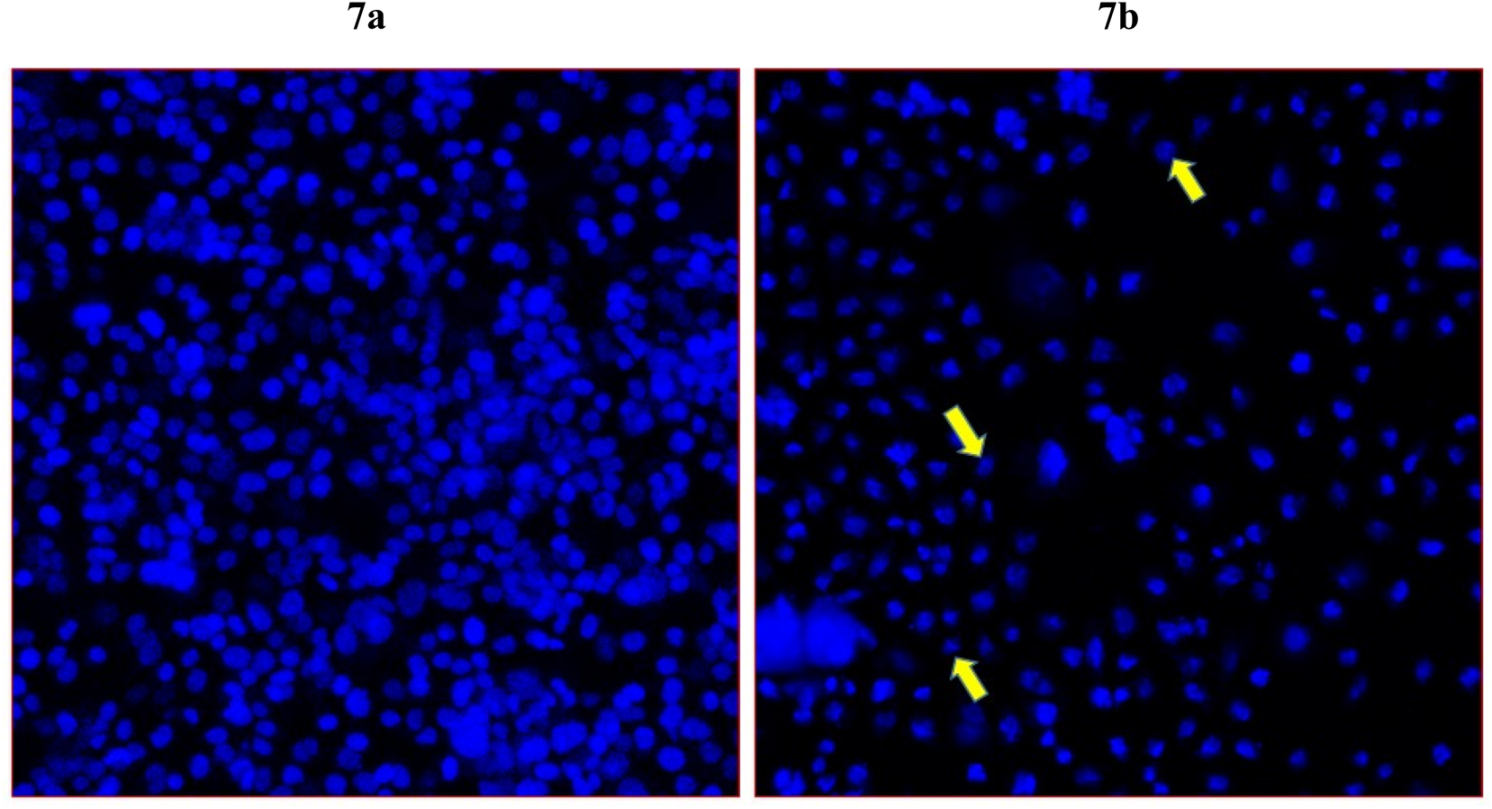
Apoptotic death of colon cancer as revealed by DAPI staining. It shows the impact of compound 7j on colon cancer cells (HCT-116). Fig. 7a is the control cells with normal morphology and cells are intact and healthy; Fig. 7b posts 48 h treatment (60 μg mL^−1^) of 7j treated cells. Arrows showing chromatin condensation, nuclear augmentation, and formation of apoptotic bodies. Magnifications 350×.

To further examine whether cancer death occur in the HCT-116 cells are due to apoptosis or programmed cell death, we have selected compound 7j which showed the highest cell death in the MTT assay for the DAPI nuclear staining. DAPI (4′,6-diamidino-2-phenylindole) which is a blue-fluorescent DNA stain used in identifying apoptosis. The treatment of compound 7j has produced a considerably high cancer cell death (Fig. 7b). In addition, we have observed chromatin condensation and formation of apoptotic bodies in the treated cells (Fig. 7b). The control cells appeared healthy without any morphological changes, as observed in treated cells (Fig. 7a).

**Fig. 8:**
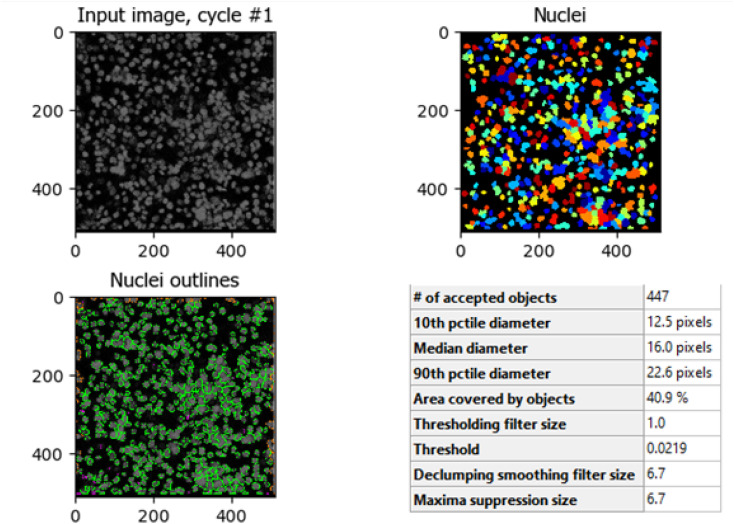
Quantitative nuclei segmentation analysis of image from untreated HCT-116 cells. The total number of nuclei detected in this image were 447.

**Fig. 9:**
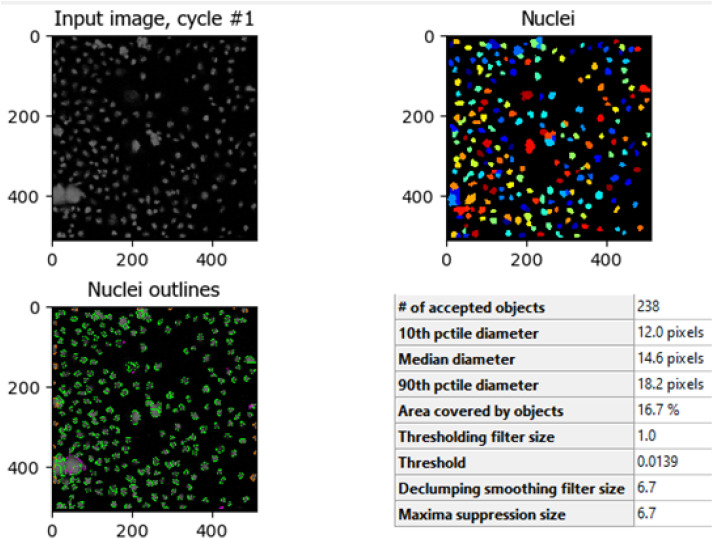
Quantitative nuclei segmentation analysis of image from HCT-116 cells treated with compound 7j for 48 hours. The total number of nuclei detected in this image were 238.

The comparison between Fig. 8 (control HCT-116 cells) and Fig. 9 (HCT-116 cells treated with 60 μg mL^−1^ of compound 7j for 48 hours) reveals significant changes in cell morphology, indicative of anti-cancer activity. In the control group (Fig. 8), 447 nuclei were detected, covering 40.9% of the image area, with a median diameter of 16.0 pixels. In contrast, the treated cells in Fig. 9 showed a marked reduction, with only 238 nuclei detected, covering 16.7% of the image area, and a smaller median diameter of 14.6 pixels. This reduction in the number of detected nuclei, along with a decrease in nuclear size, suggests cell death or inhibited cell proliferation due to the compound's cytotoxic effects. These results highlight the potential efficacy of compound 7j in reducing cell viability and inducing apoptosis in HCT-116 cells.

**Fig. 10:**
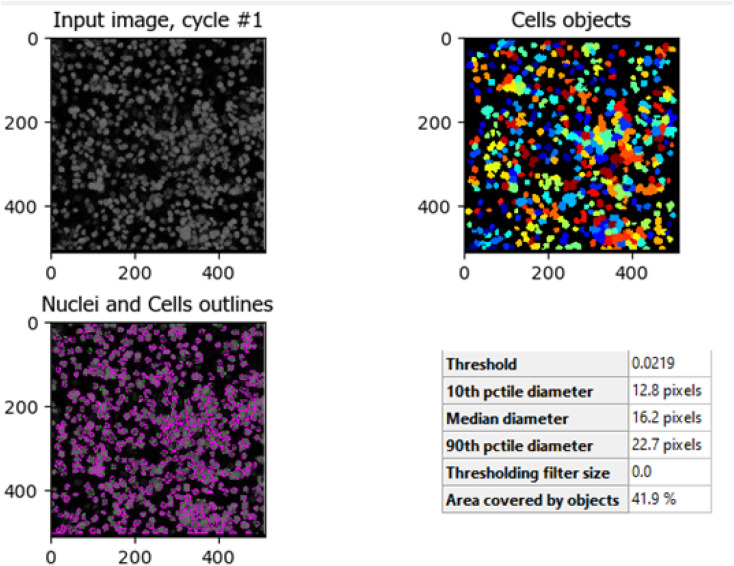
Untreated HCT-116 cells. The image shows the median diameter of the cells as 16.2 pixels, indicating healthy cell proliferation and intact cellular integrity.

**Fig. 11:**
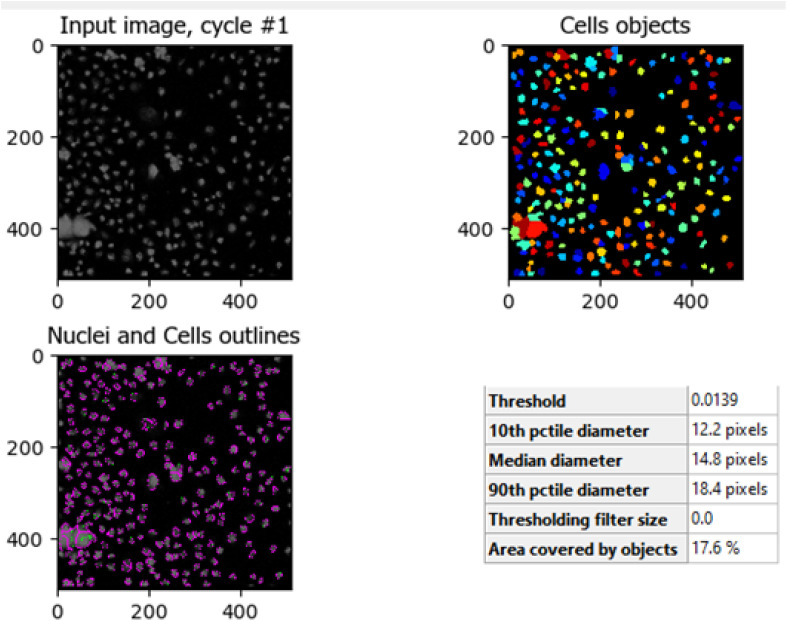
HCT-116 cells treated with 60 μg mL^−1^ of compound 7j for 48 hours. The image shows the median diameter of the cells was reduced to 14.8 pixels, reflecting the cytotoxic effect of compound 7j, which led to cell shrinkage and a reduction in cell population, indicative of apoptosis or inhibited proliferation.

The comparison between Fig. 10 (untreated HCT-116 cells) and Fig. 11 (HCT-116 cells treated with 60 μg mL^−1^ of compound 7j for 48 hours) reveals significant changes in cell population and morphology due to the anti-cancer activity of compound 7j. In the untreated cells, a higher number of cells are detected with a median diameter of 16.2 pixels, covering 41.9% of the image area, indicating robust growth and healthy cell integrity. In contrast, the treated cells show a marked reduction in cell count, with fewer, smaller cells (median diameter of 14.8 pixels) occupying only 17.6% of the image area. The shrinkage in cell size and decreased cell population suggest that compound 7j induced apoptosis or inhibited cell proliferation, leading to compromised cell integrity. This substantial reduction in cell density and size demonstrates the cytotoxic effect of compound 7j on HCT-116 cells after 48 hours of treatment.

## Conclusion

We here in report the first Michael alkylation of 3-phenylquinoxalin-2(1*H*)-one (1) with acrylic acid derivative to give only the chemoselective *N*-alkyl substituted derivatives. The ester; ethyl 3-(2-oxo-3-phenylquinoxalin-1(2*H*)-yl)propanoate (2a) reacted with hydroxyl amine and hydrazine hydrate to give the corresponding *N*-hydroxy propanamide and hydrazide. The hydrazide proved to be an excellent synthon to modify the quinoxaline structure by the reaction with aryl isothiocyanates and the reaction with amines *via* the azide coupling method. Molecular simulation results were supportive for the initial compound design and complemented the experimental results with the identification of compound 7j as the most active compound. The current research established compounds 2a and 7j as active compounds against colorectal carcinoma. While compound 7j showed highest cell death in DAPI staining and induced apoptosis. The image analysis results from HCT-116 cells treated with compound 7j further confirmed its high anti-cancer efficacy.

## Methods/experimental

### Chemical studies

#### General procedures

Solvents were purified and dried in the usual way. The boiling range of the petroleum ether used was 40–60 °C. Thin layer chromatography (TLC): silica gel 60 F_254_ plastic plates (E. Merck, layer thickness 0.2 mm) detected by UV absorption. Elemental analyses were performed on a *Flash EA-1112* instrument at the Micro analytical laboratory, Faculty of Science, Suez Canal University, Ismailia, Egypt. Melting points were determined on a Buchi 510 melting-point apparatus and the values are uncorrected. ^1^H and ^13^C NMR spectra were recorded at 400 MHz and 100 MHz, respectively (Bruker AC 400) in CDCl_3_ and DMSO solution with tetramethylsilane as an internal standard. The NMR analyses were performed at Faculty of Science, Sohag University. 3-Phenylquinoxalin-2(1*H*)-one (1) was prepared according to the described method.^50^

#### Preparation of 3-(2-oxo-3-phenylquinoxalin-1(2*H*)-yl)propanoic acid derivatives 2a–c

3-Phenylquinoxalin-2(1*H*)-one (1) (2.22 g, 10.0 mmol) was mixed with potassium carbonate (1.38 g, 10.0 mmol) and acrylic acid derivatives (40.0 mmol); ethyl acrylate, acrylamide and acrylonitrile. The reaction mixture was heated under reflux at 100–120 °C for 1 h., monitored with TLC. The reaction mixture was evaporated under reduced pressure, dissolved in ethyl acetate, washed with water and dried over sodium sulfate. The ethyl acetate solution was filtered and evaporated under reduced pressure and the resultant solid was crystalized from ethanol to give pure crystals of 3-(2-oxo-3-phenylquinoxalin-1(2*H*)-yl)propanoic acid derivatives 2a–c.

#### Ethyl 3-(2-oxo-3-phenylquinoxalin-1(2*H*)-yl)propanoate (2a)

From acrylate with 3-phenylquinoxalin-2(1*H*)-one (1). White crystals, yield 88%. Mp 65–67 °C. ^1^H NMR spectrum, (400 MHz, CDCl_3_), *δ*, ppm (*J*, Hz): 1.26 (t, *J* = 7.2 Hz, 3H, CH_3_), 2.85 (m, *J* = 6.0 Hz, 2H, CH_2_CO), 4.20 (q, *J* = 7.6 Hz, 2H, OCH_2_), 4.65 (t, *J* = 6.0 Hz, 2H, NCH_2_), 7.37–7.63 (m, 6H, Ar-H), 7.97–8.00 (m, 1H, Ar-H), 8.31–8.34 (m, 2H, Ar-H). ^13^C-NMR (100.0 MHz, CDCl_3_), *δ*, ppm: 14.1 (CH_3_), 31.8 (CH_2_CO), 38.9 (NCH_2_), 61.1 (CH_2_O), 113.3, 123.9, 128.1, 129.6, 129.9, 130.4, 130.5, 130.9, 132.2, 133.4, 135.8, 153.9 (Ar-C), 154.4, 170.9 (2CO). MS (MALDI, positive mode, matrix DHB) *m*/*z*: 345.1 (M + Na)^+^. Anal. Calcd. For C_19_H_18_N_2_O_3_ (322.13) C, 70.79; H, 5.63; N, 8.69. Found C, 70.85; H, 5.69; N, 8.74.

#### 3-(2-Oxo-3-phenylquinoxalin-1(2*H*)-yl)propanamide (2b)

From acrylamide with 3-phenylquinoxalin-2(1*H*)-one (1). White crystals, yield 92%. Mp 157–159 °C. ^1^H NMR spectrum, (400 MHz, DMSO-d6) *δ*, ppm (*J*, Hz): 3.36 (t, *J* = 6.0 Hz, 2H, CH_2_CO), 4.39 (t, *J* = 6.0 Hz, 2H, NCH_2_), 7.39–7.61 (m, 5H. Ar-H), 7.84–7.89 (m, 4H, Ar-H), 8.13 (bs, 2H, NH_2_). ^13^C-NMR (100.0 MHz, DMSO-d6), *δ*, ppm: 31.6 (CH_2_CO), 48.7 (NCH_2_), 54.3 (CH_2_OH), 114.6, 123.8, 128.1, 129.6, 130.4, 130.7, 131.3, 132.5, 132.8, 136.2, 153.4, 154.2 (Ar-C), 169.5, 171.7 (2CO). MS (MALDI, positive mode, matrix DHB) *m*/*z*: 316.35 (M + Na)^+^. Anal. Calcd. For C_17_H_15_N_3_O_2_ (293.33) C, 69.61; H, 5.15; N, 14.33. Found C, 69.67; H, 5.21; N, 14.39.

#### 3-(2-Oxo-3-phenylquinoxalin-1(2*H*)-yl)propanenitrile (2c)

From acrylonitrile with 3-phenylquinoxalin-2(1*H*)-one (1). White crystals, yield 84%. Mp 122–123 °C. ^1^H NMR spectrum, (400 MHz, CDCl_3_), *δ*, ppm (*J*, Hz): 4.22 (t, *J* = 6.2 Hz, 2H, CH_2_CN), 4.65 (t, *J* = 6.0 Hz, 2H, NCH_2_), 7.41–7.55 (m, 6H. Ar-H), 7.95–8.03 (m, 1H, Ar-H), 8.34–8.37 (m, 2H, Ar-H). ^13^C-NMR (100.0 MHz, CDCl_3_), *δ*, ppm: 22.1 (CH_2_CN), 38.7 (NCH_2_), 113.6, 119.6, 123.7, 128.2, 129.5, 130.6, 130.7, 131.1, 132.3, 133.6, 135.5, 153.9 (Ar-C), 154.6, (CO). MS (MALDI, positive mode, matrix DHB) *m*/*z*: 298.3 (M + Na)^+^. Anal. Calcd. For C_17_H_13_N_3_O (275.31) C, 74.17; H, 4.76; N, 15.26. Found C, 74.22; H, 4.81; N, 15.33.

#### *N*-Hydroxy-3-(2-oxo-3-phenylquinoxalin-1(2*H*)-yl)propanamide (3)

To a solution of ethyl 3-(2-oxo-3-phenylquinoxalin-1(2*H*)-yl)propanoate (2a) (3.22 g 10 mmol) in 30 mL ethanol (95%) was added hydroxyl amine hydrochloride (0.65 g, 10 mmol) and potassium hydroxide (0.56 g, 10 mmol). The reaction mixture was stirred at room temperature for 12 h., TLC monitored. The reaction mixture was evaporated, dissolved in ethyl acetate, washed with water and dried over sodium sulfate. The ethyl acetate solution was evaporated under reduced pressure and the resultant solid was crystalized from ethanol.

From hydroxyl amine and ethyl 3-(2-oxo-3-phenylquinoxalin-1(2*H*)-yl)propanoate (2a), white crystals, yield 79%. Mp 192–193 °C. ^1^H NMR spectrum, (400 MHz, DMSO-d6), *δ*, ppm (*J*, Hz): 3.41 (t, *J* = 6.0 Hz, 2H, CH_2_CO), 4.38 (t, *J* = 6.0 Hz, 2H, NCH_2_), 7.43–7.66 (m, 5H. Ar-H), 7.85–7.89 (m, 4H, Ar-H), 9.12 (bs, 1H, NH), 10.74 (*bs*, 1H, OH). ^13^C-NMR (100.0 MHz, DMSO-d6), *δ*, ppm: *δ*: 31.8 (CH_2_CO), 48.7 (NCH_2_), 114.4, 124.20, 128.1, 129.7, 130.1, 130.7, 131.3, 132.5, 132.8, 136.1, 153.3, 154.5 (Ar-C), 169.4, 171.2 (2CO). MS (MALDI, positive mode, matrix DHB) *m*/*z*: 332.3 (M + Na)^+^. Anal. Calcd. For C_17_H_15_N_3_O_3_ (309.33) C, 66.01; H, 4.89; N, 13.58. Found C, 66.09; H, 4.97; N, 13.64.

#### 3-(2-Oxo-3-phenylquinoxalin-1(2*H*)-yl)propanehydrazide (4)

To a solution of ethyl 3-(2-oxo-3-phenylquinoxalin-1(2*H*)-yl)propanoate (2a) (3.22 g 10 mmol) in 30 mL ethanol (95%) was added hydrazine hydrate (1.0 mL, 20 mmol). The reaction mixture was heated under reflux for 4 h. Monitored with TLC. The reaction mixture was cooled and the resultant solid crystals was obtained pure enough for analysis.

White crystals, yield 91%. Mp 161–162 °C. ^1^H NMR spectrum, (400 MHz, DMSO-d6), *δ*, ppm (*J*, Hz): 3.39 (t, *J* = 6.0 Hz, 2H, CH_2_CO), 4.21 (bs, 2H, NH_2_), 4.42 (t, *J* = 6.0 Hz, 2H, NCH_2_), 7.41–7.64 (m, 5H. Ar-H), 7.89–7.90 (m, 4H, Ar-H), 9.18 (bs, 1H, NH). ^13^C-NMR (100.0 MHz, DMSO-d6), *δ*, ppm: *δ*: 31.7 (CH_2_CO), 39.1 (NCH_2_), 114.7, 124.0, 128.3, 129.8, 130.3, 130.6, 131.1, 132.8, 132.9, 136.3, 153.3, 153.5 (Ar-C), 154.1, 169.4 (2CO). MS (MALDI, positive mode, matrix DHB) *m*/*z*: 331.3 (M + Na)^+^. Anal. Calcd. For C_17_H_16_N_4_O_2_ (308.34) C, 66.22; H, 5.23; N, 18.17. Found C, 66.26; H, 5.31; N, 18.22.

#### Preparation of 4-aryl-1-(3-(2-oxo-3-phenylquinoxalin-1(2*H*)-yl)propanoyl) thiosemicarbazides 5a–c

To a solution of 3-(2-oxo-3-phenylquinoxalin-1(2*H*)-yl)propanehydrazide (4) (3.08 g 10 mmol) in 30 mL ethanol (95%) was added aryl isothiocyanate (10 mmol). The reaction mixture was heated under reflux for 4 h monitored with TLC. The reaction mixture was cooled and the resultant solid crystals was filtered and was crystalized by ethyl acetate pet ether mixture.

#### 1-(3-(2-Oxo-3-phenylquinoxalin-1(2*H*)-yl)propanoyl)-4-phenyl thiosemicarbazide (5a)

From hydrazide 3 with phenyl isothiocyanate. White crystals, yield 82%. Mp 161–172 °C. ^1^H NMR spectrum, (400 MHz, DMSO-d6), *δ*, ppm (*J*, Hz): 3.41 (t, *J* = 6.0 Hz, 2H, CH_2_CO), 4.41 (t, *J* = 6.0 Hz, 2H, NCH_2_), 7.14–7.16 (m, 1H, Ar-H) 7.42–7.63 (m, 7H. Ar-H), 7.88–7.91 (m, 6H, Ar-H), 8.17 (bs, 1H, NH), 9.77 (bs, 1H, NH), 10.93 (bs, 1H, NH). ^13^C-NMR (100.0 MHz, DMSO-d6), *δ*, ppm: 31.6 (CH_2_CO), 48.6 (NCH_2_), 54.7 (CH_2_OH), 114.6, 124.2, 127.1, 128.3, 128.8, 129.3, 129.7, 130.4, 130.5, 131.3, 132.7, 132.8, 136.1, 137.4, 153.5, 154.2 (Ar-C), 169.5, 170.3 (2CO), 177.4 (CS). MS (MALDI, positive mode, matrix DHB) *m*/*z*: 466.5 (M + Na)^+^. Anal. Calcd. For C_24_H_21_N_5_O_2_S (443.53) C, 64.99; H, 4.77; N, 15.79; S, 7.23. Found C, 65.07; H, 4.84; N, 15.88; S, 7.27.

#### 4-(4-Methoxyphenyl)-1-(3-(2-oxo-3-phenylquinoxalin-1(2*H*)-yl)propanoyl) thiosemicarbazide (5b)

From hydrazide 3 with 4-methoxyphenyl isothiocyanate. White crystals, yield 87%. Mp 188–189 °C. ^1^H NMR spectrum, (400 MHz, DMSO-d6), 3.38 (t, *J* = 6.0 Hz, 2H, CH_2_CO), 3.78 (s, 3H, OMe), 4.41 (t, *J* = 6.0 Hz, 2H, NCH_2_), 7.13–7.15 (m, 1H, Ar-H) 7.41–7.60 (m, 7H. Ar-H), 7.85–7.92 (m, 6H, Ar-H), 8.19 (bs, 1H, NH), 9.81 (bs, 1H, NH), 10.89 (bs, 1H, NH). ^13^C-NMR (100.0 MHz, DMSO-d6), *δ*, ppm: 31.7 (CH_2_CO), 48.6 (NCH_2_), 54.7 (CH_2_OH), 58.4 (OMe), 114.4, 124.1, 127.3, 128.2, 128.6, 129.1, 129.6, 130.2, 130.6, 131.4, 132.5, 132.9, 136.2, 137.6, 153.4, 154.2 (Ar-C), 169.6, 170.4 (2CO), 178.6 (CS). MS (MALDI, positive mode, matrix DHB) *m*/*z*: 496.5 (M + Na)^+^. Anal. Calcd. For C_25_H_23_N_5_O_3_S (473.55) C, 63.41; H, 4.90; N, 14.79; S, 6.77. Found C, 63.49; H, 4.97; N, 14.85; S, 6.83.

#### 1-(3-(2-Oxo-3-phenylquinoxalin-1(2*H*)-yl)propanoyl)-4-(4-tolyl) thiosemicarbazide (5c)

From hydrazide 3 with 4-methylphenyl isothiocyanate. White crystals, yield 79%. Mp 173–174 °C. ^1^H NMR spectrum, (400 MHz, DMSO-d6), *δ*, ppm (*J*, Hz): 1.98 (s, 3H, CH_3_), 3.37 (t, *J* = 6.0 Hz, 2H, CH_2_CO), 4.39 (t, *J* = 6.0 Hz, 2H, NCH_2_), 7.11–7.14 (m, 1H, Ar-H) 7.43–7.62 (m, 7H. Ar-H), 7.84–7.91 (m, 6H, Ar-H), 8.15 (bs, 1H, NH), 9.83 (bs, 1H, NH), 10.86 (bs, 1H, NH). ^13^C-NMR (100.0 MHz, DMSO-d6), *δ*, ppm: 19.3 (CH_3_), 31.5 (CH_2_CO), 48.4 (NCH_2_), 54.8 (CH_2_OH), 114.1, 124.3, 127.5, 128.4, 128.7, 129.3, 129.7, 130.3, 130.7, 131.6, 132.5, 132.8, 136.3, 137.4, 153.2, 154.4 (Ar-C), 169.5, 170.7 (2CO), 178.4 (CS). MS (MALDI, positive mode, matrix DHB) *m*/*z*: 480.5 (M + Na)^+^. Anal. Calcd. For C_25_H_23_N_5_O_2_S (457.55) C, 65.63; H, 5.07; N, 15.31; S, 7.01. Found C, 65.71; H, 5.16; N, 15.37; S, 7.09.

#### Preparation of *N*-alkyl-3-(2-oxo-3-phenylquinoxalin-1(2*H*)-yl)propanamides 7a–j

A solution of 3-(2-oxo-3-phenylquinoxalin-1(2*H*)-yl)propanehydrazide (4) (0.308 g 1.0 mmol) in 15 mL 1 N HCl and 5 mL acetic acid was kept with constant stirring in an ice bath at −5 °C. To this solution was added cold solution of sodium nitrite (0.1 g, 1.5 mmol) in 2 mL water, portion wise with constant stirring. The reaction mixture was further stirred at −5 °C for 1 h, extracted with ethyl acetate and washed with sodium carbonate (10 mL, 1 N), HCl (10 mL, 1 N) and water (10 mL) and finally dried over sodium sulfate to afford the *in situ* generated ethyl acetate solution of azide 6. Cold ethyl acetate azide solution 6 was rapidly added to a cold ethyl acetate solution of amines; propyl amine, butyl amine, isopropyl amine, allyl amine, benzyl amine, cyclohexyl amine, morpholine, piperidine, pyrrolidine and β-naphthylethylenediamine and was kept in the freezer for 12 h. The ethyl acetate solution was left at room temperature for another 12 h then washed with water and dried over sodium sulfate. The ethyl acetate solution was filtered, evaporated and crystalized from ethyl acetate pet ether mixture.

#### *N*-propyl-3-(2-oxo-3-phenylquinoxalin-1(2*H*)-yl)propanamide (7a)

From *n*-propylamine and the azide 6. White crystals, yield 72%. Mp 111–112 °C. ^1^H NMR spectrum, (400 MHz, CDCl_3_), *δ*, ppm (*J*, Hz): 0.86–0.95 (m, 3H, CH_3_), 1.41–1.59 (m, 2H, CH_2_), 2.67–2.72 (m, 2H, CH_2_CO), 3.16–3.22 (m, 2H, NHCH_2_), 4.62–4.67 (m, 2H, NCH_2_), 6.19 (bs, 1H, NH), 7.36–7.60 (m, 6H, Ar-H), 7.94–7.97 (m, 1H, Ar-H), 8.26–8.29 (m, 2H, Ar-H). ^13^C-NMR (100.0 MHz, CDCl_3_), *δ*, ppm: 11.6 (CH_3_), 22.6 (CH_2_), 34.2 (CH_2_CO), 39.6 (NCH_2_), 41.4 (NHCH_2_), 113.9, 124.0, 128.1, 129.5, 130.4, 130.6, 130.7, 132.1, 133.4, 135.8, 153.7 (Ar-C), 154.5, 169.9 (2CO). MS (MALDI, positive mode, matrix DHB) *m*/*z*: 358.4 (M + Na)^+^. Anal. Calcd. For C_20_H_21_N_3_O_2_ (335.41) C, 71.62; H, 6.31; N, 12.53. Found C, 71.70; H, 6.39; N, 12.61.

#### *N*-Butyl-3-(2-oxo-3-phenylquinoxalin-1(2*H*)-yl)propanamide (7b)

From *n*-butylamine and the azide 6. White crystals, yield 76%. Mp 124–125 °C. ^1^H NMR spectrum, (400 MHz, CDCl_3_), *δ*, ppm (*J*, Hz): 0.86–0.91 (m, 3H, CH_3_), 1.27–1.47 (m, 4H, 2CH_2_), 2.64–2.69 (m, 2H, CH_2_CO), 3.21–3.22 (m, 2H, NHCH_2_), 4.57–4.62 (m, 2H, NCH_2_), 6.56 (bs, 1H, NH), 7.32–7.56 (m, 6H, Ar-H), 7.90–7.93 (m, 1H, Ar-H), 8.22–8.25 (m, 2H, Ar-H). ^13^C-NMR (100.0 MHz, CDCl_3_), *δ*, ppm: 13.7 (CH_3_), 20.0 (CH_2_), 23.1 (CH_2_), 31.5 (CH_2_CO), 39.4 (NCH_2_), 39.6 (NHCH_2_), 113.9, 123.9, 128.0, 129.4, 130.3, 130.6, 130.7, 132.1, 133.3, 135.8, 153.7 (Ar-C), 154.5, 169.9 (2CO). MS (MALDI, positive mode, matrix DHB) *m*/*z*: 372.4 (M + Na)^+^. Anal. Calcd. For C_21_H_23_N_3_O_2_ (349.43) C, 72.18; H, 6.63; N, 12.03. Found C, 72.23; H, 6.72; N, 12.11.

#### *N*-Isopropyl-3-(2-oxo-3-phenylquinoxalin-1(2*H*)-yl)propanamide (7c)

From isopropylamine and the azide 6. White crystals, yield 74%. Mp 108–109 °C. ^1^H NMR spectrum, (400 MHz, CDCl_3_), *δ*, ppm (*J*, Hz): 1.11 (d, *J* = 8.8 Hz, 6H, 2CH_3_), 2.66–2.71 (m, 2H, CH_2_CO), 4.02–4.13 (m, 1H, NHCH), 4.63–4.68 (m, 2H, NCH_2_), 5.70 (bs, 1H, NH), 7.37–7.62 (m, 6H, Ar-H), 7.96–7.99 (m, 1H, Ar-H), 8.30–8.34 (m, 2H, Ar-H). ^13^C-NMR (100.0 MHz, CDCl_3_), *δ*, ppm: 22.6 (2CH_3_), 34.4 (CH_2_CO), 39.6 (NCH_2_), 41.8 (NCH), 113.9, 114.0, 124.1, 128.1, 129.6, 130.4, 130.6, 130.8, 132.2, 133.3, 135.7, 153.7 (Ar-C), 154.6, 169.0 (2CO). MS (MALDI, positive mode, matrix DHB) *m*/*z*: 358.4 (M + Na)^+^. Anal. Calcd. For C_20_H_21_N_3_O_2_ (335.41) C, 71.62; H, 6.31; N, 12.53. Found C, 71.69; H, 6.37; N, 12.60.

#### *N*-Allyl-3-(2-oxo-3-phenylquinoxalin-1(2*H*)-yl)propanamide (7d)

From allylamine and the azide 6. White crystals, yield 88%. Mp 112–113 °C. ^1^H NMR spectrum, (400 MHz, CDCl_3_), *δ*, ppm (*J*, Hz): 2.71–2.76 (m, 2H, CH_2_CO), 3.86–3.91 (m, 2H, NCH_2_), 4.64–4.69 (m, 2H, CH_2_), 5.10–5.19 (m, 2H, NCH_2_), 5.72–5.83 (m, 1H, CH), 6.13 (bs, 1H, NH), 7.37–7.61 (m, 6H, Ar-H), 7.97–8.00 (m, 1H, Ar-H), 8.27–8.30 (m, 2H, Ar-H). ^13^C-NMR (100.0 MHz, CDCl_3_), *δ*, ppm: 34.1 (CH_2_CO), 39.5 (NCH_2_), 42.1 (NCH_2_), 113.8, 116.7, 124.1, 128.1, 129.5, 130.5, 130.7, 130.9, 132.1, 133.4, 135.7, 153.7 (Ar-C), 154.6, 169.7 (2CO). MS (MALDI, positive mode, matrix DHB) *m*/*z*: 356.3 (M + Na)^+^. Anal. Calcd. For C_20_H_19_N_3_O_2_ (333.39) C, 72.05; H, 5.74; N, 12.60. Found C, 72.11; H, 5.80; N, 12.57.

#### *N*-Benzyl-3-(2-oxo-3-phenylquinoxalin-1(2*H*)-yl)propanamide (7e)

From benzylamine and the azide 6. White crystals, yield 78%. Mp 146–147 °C. ^1^H NMR spectrum, (400 MHz, CDCl_3_), *δ*, ppm (*J*, Hz): 2.71–2.76 (m, 2H, CH_2_CO), 4.41–4.44 (m, 2H, CH_2_Ph), 4.63–4.69 (m, 2H, NCH_2_), 6.44 (bs, 1H, NH), 7.26–7.31 (m, 3H, Ar-H), 7.37–7.59 (m, 5H, Ar-H), 7.79–7.82 (m, 2H, Ar-H), 8.24–8.28 (m, 2H, Ar-H), 8.31–8.41 (m, 2H, Ar-H). ^13^C-NMR (100.0 MHz, CDCl_3_), *δ*, ppm: 34.3 (CH_2_CO), 39.2 (NCH_2_), 42.6 (NCH_2_), 113.4, 114.6, 124.3, 127.2, 127.7, 128.3, 128.4, 128.7, 129.2, 130.1, 130.6, 130.9, 132.8, 133.1, 133.6, 135.6, 153.4 (Ar-C), 154.8, 169.2 (2CO). MS (MALDI, positive mode, matrix DHB) *m*/*z*: 406.4 (M + Na)^+^. Anal. Calcd. For C_24_H_21_N_3_O_2_ (383.45) C, 75.18; H, 5.52; N, 10.96. Found C, 75.24; H, 5.59; N, 10.91.

#### *N*-Cyclohexyl-3-(2-oxo-3-phenylquinoxalin-1(2*H*)-yl)propanamide (7f)

From cyclohexylamine and the azide 6. White crystals, yield 71%. Mp 116–118 °C. ^1^H NMR spectrum, (400 MHz, CDCl_3_), *δ*, ppm (*J*, Hz): 1.06–1.16 (m, 2H, CH_2_), 1.32–1.38 (m, 4H, 2CH_2_), 1.65–1.87 (m, 4H, 2CH_2_), 2.67–2.71 (m, 2H, CH_2_CO), 4.63–4.68 (m, 3H, NCH & NCH_2_), 7.37–7.62 (m, 6H, Ar-H & NH), 7.96–8.00 (m, 2H, Ar-H), 8.30–8.33 (m, 2H, Ar-H). ^13^C-NMR (100.0 MHz, CDCl_3_), *δ*, ppm: 24.8 (2CH_2_), 25.4 (CH_2_), 32.9 (2CH_2_), 34.4 (CH_2_CO), 39.6 (NCH_2_), 48.6 (NHCH), 113.9, 124.1, 128.1, 129.5, 130.5, 130.6, 130.8, 132.2, 133.3, 135.7, 153.7 (Ar-C), 154.6, 168.9 (2CO). MS (MALDI, positive mode, matrix DHB) *m*/*z*: 398.4 (M + Na)^+^. Anal. Calcd. For C_23_H_25_N_3_O_2_ (375.47) C, 73.57; H, 6.71; N, 11.19. Found C, 73.62; H, 6.79; N, 11.25.

#### 1-(3-Morpholino-3-oxopropyl)-3-phenylquinoxalin-2(1*H*)-one (7g)

From morpholine and the azide 6. White crystals, yield 79%. Mp 155–156 °C. ^1^H NMR spectrum, (400 MHz, CDCl_3_), *δ*, ppm (*J*, Hz): 2.82–2.82 (m, 2H, CH_2_CO), 3.47–3.50 (m, 2H, NCH_2_), 3.62–3.66 (m, 6H, NCH_2_ & 2OCH_2_), 4.63–4.69 (m, 2H, CH_2_), 7.38–7.64 (m, 5H, Ar-H), 7.97–8.00 (m, 2H, Ar-H), 8.28–8.33 (m, 2H, Ar-H). ^13^C-NMR (100.0 MHz, CDCl_3_), *δ*, ppm: 30.4 (CH_2_CO), 39.4 (NCH_2_), 42.0 (NCH_2_), 45.9 (NCH_2_), 66.6 (OCH_2_), 66.8 (OCH_2_), 113.5, 123.9, 128.1, 128.2, 129.5, 130.5, 130.8, 130.9, 132.2, 133.4, 135.7, 153.9 (Ar-C), 154.6, 168.7 (2CO). MS (MALDI, positive mode, matrix DHB) *m*/*z*: 486.4 (M + Na)^+^. Anal. Calcd. For C_21_H_21_N_3_O_3_ (363.42) C, 69.41; H, 5.82; N, 11.56. Found C, 69.49; H, 5.94; N, 11.62.

#### 1-(3-Oxo-3-(piperidin-1-yl)propyl)-3-phenylquinoxalin-2(1*H*)-one (7h)

From piperidine and azide 6. White crystals, yield 81%. Mp 141–142 °C. ^1^H NMR spectrum, (400 MHz, CDCl_3_), *δ*, ppm (*J*, Hz): 1.50–1.63 (m, 6H, 3CH_2_), 2.81–2.86 (m, 2H, CH_2_CO), 3.37–3.40 (m, 2H, NCH_2_), 3.56–3.59 (m, 2H, NCH_2_), 4.62–4.68 (m, 2H, NCH_2_), 7.35–7.59 (m, 5H, Ar-H), 7.95–7.98 (m, 2H, Ar-H), 8.28–8.32 (m, 2H, Ar-H). ^13^C-NMR (100.0 MHz, CDCl_3_), *δ*, ppm: 24.4 (CH_2_), 25.5 (CH_2_), 26.4 (CH_2_), 30.5 (CH_2_CO), 39.6 (NCH_2_), 42.7 (NCH_2_), 46.6 (NCH_2_), 113.7, 123.8, 128.1, 129.5, 130.4, 130.6, 130.9, 132.3, 133.4, 135.8, 153.8 (Ar-C), 154.6, 168.2 (2CO). MS (MALDI, positive mode, matrix DHB) *m*/*z*: 384.4 (M + Na)^+^. Anal. Calcd. For C_22_H_23_N_3_O_2_ (361.45) C, 73.11; H, 6.41; N, 11.63. Found C, 73.20; H, 6.49; N, 11.67.

#### 1-(3-Oxo-3-(pyrrolidin-1-yl)propyl)-3-phenylquinoxalin-2(1*H*)-one (7i)

From pyrrolidine and the azide 6. White crystals, yield 78%. Mp 116–117 °C. ^1^H NMR spectrum, (400 MHz, CDCl_3_), *δ*, ppm (*J*, Hz): 1.82–2.01 (m, 4H, 2CH_2_), 2.78–2.83 (m, 2H, CH_2_CO), 3.42 (t, *J* = 8.4 Hz, 2H, NCH_2_), 3.51 (t, *J* = 8.4 Hz, 2H, NCH_2_), 4.66–4.72 (m, 2H, NCH_2_), 7.36–7.62 (m, 6H, Ar-H), 7.96–7.99 (m, 1H, Ar-H), 8.29–8.32 (m, 2H, Ar-H). ^13^C-NMR (100.0 MHz, CDCl_3_), *δ*, ppm: 24.4 (CH_2_), 26.0 (CH_2_), 32.0 (CH_2_CO), 39.1 (NCH_2_), 45.9 (NCH_2_), 46.9 (NCH_2_), 113.9, 123.5, 123.9 128.1, 129.6, 130.4, 130.6, 130.7, 132.3, 133.3, 135.8, 153.9 (Ar-C), 154.6, 168.7 (2CO). MS (MALDI, positive mode, matrix DHB) *m*/*z*: 370.4 (M + Na)^+^. Anal. Calcd. For C_21_H_21_N_3_O_2_ (347.42) C, 72.60; H, 6.09; N, 12.10. Found C, 72.66; H, 6.15; N, 12.14.

#### *N*-(2-(Naphthalen-2-ylamino)ethyl)-3-(2-oxo-3-phenylquinoxalin-1(2*H*)-yl)propanamide (7j)

From β-naphthylethylenediamine and the azide 6. White crystals, yield 84%. Mp 171–172 °C. ^1^H NMR spectrum, (400 MHz, CDCl_3_), *δ*, ppm (*J*, Hz): 2.69–2.77 (m, 2H, CH_2_CO), 3.42–3.45 (m, 2H, NHCH_2_), 3.65–3.71 (m, 2H, NCH_2_), 4.62–4.66 (m, 2H, NCH_2_), 6.86 (bs, 1H, NH), 7.15 (bs, 1H, NH), 7.27–7.55 (m, 8H, Ar-H), 7.75–7.80 (m, 3H, Ar-H), 7.93–7.98 (m, 3H, Ar-H), 8.18–8.22 (m, 2H, Ar-H). ^13^C-NMR (100.0 MHz, CDCl_3_), *δ*, ppm: 34.2 (CH_2_CO), 38.7 (NCH_2_), 39.5 (NCH_2_), 42.7 (NCH_2_), 113.7, 120.2, 123.5, 124.1, 125.2, 125.9, 126.4, 128.1, 128.6, 129.4, 130.4, 130.7, 132.0, 133.4, 134.3, 135.7, 141.9, 153.7 (Ar-C), 154.7, 171.3 (2CO). MS (MALDI, positive mode, matrix DHB) *m*/*z*: 485.5 (M + Na)^+^. Anal. Calcd. For C_29_H_26_N_4_O_2_ (462.55) C, 75.30; H, 5.67; N, 12.11. Found C, 75.38; H, 5.72; N, 12.21.

### Biological studies

#### Crystal structures

The X-ray coordinates of MMP-2 and MMP-9 (PDB IDs: 8H78, and 4H3X, respectively) were extracted from the Research Collaboratory for Structural Bioinformatics (RCSB) Protein Data Bank (PDB).^51,52^

#### Protein preparation

The PDB structures were prepared for docking by the Protein Preparation Workflow provided by Schrodinger, LLC in 2018, which is accessible within the Maestro software (version 11.8). The preparation and minimization process was conducted at a pH of 7.4, altering ionization states as necessary. Polar hydrogens were added, whereas non-essential water molecules were removed from the structures. The receptors were fine-tuned in Maestro 11.8 using the OPLS3 force field before docking. The optimization and reduction of the ligand–protein complexes were conducted in the last stage using the OPLS3 force field, with a preset RMSD value of 0.30 Å for non-hydrogen atoms.^53^ The receptor grids were generated utilizing the synthesized proteins, and the docking grids were placed at the core of the attached ligand for each receptor. A receptor grid was established using a 1.00 van der Waals radius. The van der Waals (vdW) radius is adjusted by a scaling factor and partial charges are limited to a cutoff of 0.25. The binding sites were restricted inside a 20 Å^3^ grid box using default settings and no limitations.

#### Ligand library preparation

The ligands to be docked were developed using LigPrep, a software tool available in Maestro 11.8 (LigPrep, version 11.8; Schrodinger, LLC: New York, NY, 2017). The ligands' 3D structures were designed and fine-tuned for docking by determining the most probable ionization states at a pH of 7 ± 1 while preserving the initial ionization state. Therapies are given to the structures in a series at this step. Geometry is ultimately enhanced using the OPLS3 force field. The generated conformations were used as the starting input structures for the docking procedure.

#### Validation of molecular docking

The validation of the molecular docking method included assessing the ability of Maestro Glide to forecast conformations that closely resemble the experimental ones, as documented in earlier studies.^54–56^ The crystallographic ligands were docked into their corresponding targets using the identical docking method as the potential ligands. The conformation with the lowest binding energy for each target was determined when docked. The selected pose was matched with the conformation displayed in the crystallographic structure by utilizing Maestro's structure superimposition capability. The RMSD of the alignments was subsequently computed.

#### Molecular docking

The ligands underwent docking procedures utilizing the extra precision mode (XP) without any limitations. The parameters utilized were a van der Waals (vdw) radius scaling factor of 0.80 and a partial charge cut-off of 0.15. The GlideScore, a component of the Glide software, was used to evaluate the binding strength and prioritize ligands. The XP attitude Rank was used to determine and select the best docked pose for each ligand. The compounds were extensively analyzed, considering binding scores and doing a rigorous review of all binding interactions, especially with the Zn cation.

### Anti-cancer *in vitro* analysis

Human colorectal carcinoma (HCT-116) cells were purchased from ATCC, USA, and were used to examine the effect of compounds; 2a, 4, 7a, 7g, 7d, 7h, 7e, 7b, 7c, 7f, 7j. The MTT assay was followed as per previously published studies.^57^ The cells were seeded in the 96 well plates containing DMEM media and kept in a CO_2_ incubator for 24 hours. Once reach to 70–80% confluence, HCT-116 cells were treated with compounds 2a, 4, 7a, 7g, 7d, 7h, 7e, 7b, 7c, 7f, 7j in dosage range of 5.0 μg to 60 μg mL^−1^ for 48 hours and processed for MTT assay.^58,59^ In the control cells, the compounds 2a, 4, 7a, 7g, 7d, 7h, 7e, 7b, 7c, 7f, 7j were not added. The HCT-116 cells were treated with cisplatin (5.0 μg mL^−1^ to 60 μg mL^−1^) for 48 hours. Thereafter cells were exposed to MTT (5.0 mg mL^−1^) for 4 hours and media was replaced with DMSO. The plates were examined under a Plate Reader supplied by Bio-Tek instruments; USA at 570 nm wavelength and optical density (OD) was obtained to calculate the percentage of cell viability. The data presented in the graph as mean ± standard deviation obtained from triplicates and statistically evaluated by GraphPad Version 6.0 Prism Software USA.

#### Apoptotic DAPI nuclear staining

To examine the impact of the treatment on the nucleus of HCT-116 cancer cells, we selected the compound which has shown the highest cancer cell death due to treatment. We treated the cells with compound 7j with a 60 μg mL^−1^ dose for 48 hours. After that cells were treated with paraformaldehyde and then labeled with DAPI dye. The blue fluorescent staining was examined under Confocal Scanning Microscope (Zeiss, Germany).

#### Image analysis using CellProfiler

##### a. Sample preparation and treatment

HCT-116 colorectal cancer cells were cultured and treated with 60 μg mL^−1^ of compound 7j for 48 hours. Untreated control cells were also prepared for comparison. After treatment, cells were fixed and stained with DAPI (4′,6-diamidino-2-phenylindole) to visualize the nuclei.

##### b. Image acquisition

Images of the stained cells were captured using a fluorescence microscope with a DAPI filter set, ensuring uniform settings (exposure time, magnification, *etc.*) for both untreated (control) and treated cells. Images were saved in high-resolution formats compatible with CellProfiler, such as .tiff or .png, for further analysis.

##### c. Image analysis pipeline in CellProfiler

The following steps were followed using CellProfiler software to quantify the impact of compound 7j on the HCT-116 cells.

#### Image loading and preprocessing

The acquired images were loaded into CellProfiler using the “Images” module. The control (untreated) and treated cell images were processed separately. Preprocessing was conducted to improve image quality, which involved adjusting the contrast and removing background noise, using the “RescaleIntensity” module.

#### Identification of primary objects (nuclei detection)

The “IdentifyPrimaryObjects” module was used to detect the nuclei in both untreated and treated samples. The size of the nuclei was specified, and automatic thresholding (Otsu method) was applied to distinguish the nuclei from the background. Post-segmentation, overlapping or clumped nuclei were separated using declumping parameters, such as smoothing filters and maxima suppression. Quantitative data such as the number of detected nuclei, nuclear area, and shape features were measured for further analysis.

#### Identification of secondary objects (cells)

The “IdentifySecondaryObjects” module was used to detect the cell boundaries based on the nuclei identified in the previous step. A propagation method was applied to detect the full cell area around the nucleus, and quantitative measurements such as cell count, cell size, and cell area were recorded.

#### Measurement of cell properties

The “MeasureObjectSizeShape” module was applied to calculate the size, shape, and area of both the nuclei and cells. The “MeasureObjectIntensity” module was used to measure the intensity of DAPI staining, helping to assess nuclear integrity and potential apoptosis-related changes.

##### d. Data analysis and interpretation

Quantitative results from the CellProfiler image analysis were analyzed to determine the effect of compound 7j on cell proliferation and morphology. A significant reduction in the number of detected cells, along with smaller cell and nuclear sizes, in the treated samples indicated cytotoxicity and apoptosis induction by compound 7j. These findings were corroborated by visual inspections of the segmented images, which displayed fewer, smaller, and less intact cells in the treated samples compared to the controls.

## Abbreviations

DAPI: 4′, 6-Diamidino-2-phenylindole
HCT-116: Human colorectal cancer cell lines
CRC: Colorectal cancer
MMPs: Matrix metalloproteinases
ECM: Extracellular matrix
MMPIs: Matrix metalloproteinases inhibitors
ZBGs: Zinc-binding groups
DMF: Dimethyl formamide
IC50: Inhibitory concentration
DMSO: Dimethyl sulfoxide
RCSB: Research Collaboratory for Structural Bioinformatics
PDB: Protein Data Bank
vdW: van der Waals
RMSD: Root Mean Square Deviation

## Data availability

All data generated or analysed during this study are included in this published article [and its ESI files†]. The crystal structures analysed during the current study are available in the PDB database, [PDB IDs: 8H78 & 4H3X]. The MTT assay and DAPI data will be available upon request.

## Author contributions

G. M. S, S. M. R, I. A. A, W. F, M. S. A, A. H. K written the main building of the manuscript. A. H. A., M. F. A, F. H. P, F. A. K described the chemical and biological analysis. A. H. A. A, M. A draw the tables and figures. All authors take part in the revision and practical part of the whole manuscript.

## Conflicts of interest

The authors declare that they have no competing interests.

## Supplementary Material

RA-014-D4RA06822J-s001

## References

[cit1] Sung H., Ferlay J., Siegel R. L., Laversanne M., Soerjomataram I., Jemal A. (2021). *et al.*, Global Cancer Statistics 2020: GLOBOCAN Estimates of Incidence and Mortality Worldwide for 36 Cancers in 185 Countries. Ca-Cancer J. Clin..

[cit2] Sugreev V., Kousik K., Nilanjan G., Sayantan J., Snehasikta S. (2014). Matrix metalloproteinases and gastrointestinal cancers: impacts of dietary antioxidants. World J. Biol. Chem..

[cit3] Bramhall S. R., Schulz J., Nemunaitis J., Brown P. D., Baillet M., Buckels J. A. (2002). A Double-Blind Placebo-Controlled, Randomised Study Comparing Gemcitabine and Marimastat With Gemcitabine and Placebo as First Line Therapy in Patients With Advanced Pancreatic Cancer. Br. J. Cancer.

[cit4] Sparano J. A., Bernardo P., Stephenson P., Gradishar W. J., Ingle J. N., Zucker S. (2004). *et al.*, Randomized Phase III Trial of Marimastat *Versus* Placebo in Patients With Metastatic Breast Cancer Who Have Responding or Stable Disease After First-Line Chemotherapy: Eastern Cooperative Oncology Group Trial E2196. J. Clin. Oncol..

[cit5] Hirte H., Vergote I. B., Jeffrey J. R., Grimshaw R. N., Coppieters S., Schwartz B. (2006). *et al.*, A Phase III Randomized Trial of BAY 12-9566 (Tanomastat) as Maintenance Therapy in Patients With Advanced Ovarian Cancer Responsive to Primary Surgery and Paclitaxel/Platinum Containing Chemotherapy: A National Cancer Institute of Canada Clinical Trials Group Study. Gynecol. Oncol..

[cit6] Winer A., Adams S., Mignatti P. (2018). Matrix Metalloproteinase Inhibitors in Cancer Therapy: Turning Past Failures Into Future Successes. Mol. Cancer Ther..

[cit7] Shah M. A., Starodub A., Sharma S., Berlin J., Patel M., Wainberg Z. A. (2018). *et al.*, Andecaliximab/GS-5745 Alone and Combined With Mfolfox in Advanced Gastric and Gastroesophageal Junction Adenocarcinoma: Results From a Phase I Study. Clin. Cancer Res..

[cit8] Pezeshkian Z., Nobili S., Peyravian N., Shojaee B., Nazari H., Soleimani H., Asadzadeh-Aghdaei H., Ashrafian M. B., Nazemalhosseini-Mojarad E., Mini E. (2021). Insights into the Role of Matrix Metalloproteinases in Precancerous Conditions and in Colorectal Cancer. Cancers.

[cit9] Barabás L., Hritz I., István G., Tulassay Z., Herszényi L. (2021). The Behavior of MMP-2, MMP-7, MMP-9, and Their Inhibitors TIMP-1 and TIMP-2 in Adenoma-Colorectal Cancer Sequence. Dig. Dis..

[cit10] Salem N., Kamal I., Al-Maghrabi J., Abuzenadah A., Peer-Zada A. A., Qari Y., Al-Ahwal M., Al-Qahtani M., Buhmeida A. (2016). High expression of matrix metalloproteinases: MMP-2 and MMP-9 predicts poor survival outcome in colorectal carcinoma. Future Oncol..

[cit11] Chen H., Ye Y., Yang Y., Zhong M., Gu L., Han Z., Qiu J., Liu Z., Qiu X., Zhuang G. (2020). TIPE-mediated up-regulation of MMP-9 promotes colorectal cancer invasion and metastasis through MKK-3/p38/NF-kB pro-oncogenic signaling pathway. Signal Transduction Targeted Ther..

[cit12] Walter L., Canup B., Pujada A., Bui T. A., Arbasi B., Laroui H., Merlin D., Garg P. (2020). Matrix metalloproteinase 9 (MMP9) limits reactive oxygen species (ROS) accumulation and DNA damage in colitis-associated cancer. Cell Death Dis..

[cit13] Rao B. G. (2005). Recent developments in the design of specific matrix
metalloproteinase inhibitors aided by structural and computational studies. Curr. Pharm. Des..

[cit14] Stephen B., Samy O. M., Rafael F., Shahriar M. (2004). Quest for selectivity in inhibition of matrix metalloproteinases. Curr. Top. Med. Chem..

[cit15] Breuer E., Frant J., Reich R. (2005). Recent non-hydroxamate matrix metalloproteinase inhibitors. Expert Opin. Ther. Pat..

[cit16] Jacobsen J. A., Jourden J. L. M., Miller M. T., Cohen S. M. (2010). To bind zinc or not to bind zinc: an examination of innovative approaches to improved metalloproteinase inhibition. Biochim. Biophys. Acta.

[cit17] Dublanchet A.-C., Ducrot P., Andrianjara C., O'Gara M., Morales R., Compere D., Denis A., Blais S., Cluzeau P., Courte K., Hamon J., Moreau F., Prunet M. L., Tertre A. (2005). Structure-based design and synthesis of novel non-zinc chelating MMP-12 inhibitors. Bioorg. Med. Chem. Lett..

[cit18] Ayoup M. S., Fouad M. A., Abdel-Hamid H., Ramadan E. S., Abu-Serie M. M., Noby A., Teleb M. (2020). Battle tactics against MMP-9; discovery of novel nonhydroxamate MMP-9 inhibitors endowed with PI3K/AKT signaling attenuation and caspase 3/7 activation *via* Ugi bis-amide synthesis. Eur. J. Med. Chem..

[cit19] Mondal S., Adhikari N., Banerjee S., Abdul Amin S., Jha T. (2020). Matrix metalloproteinase-9 (MMP-9) and its inhibitors in cancer: a minireview. Eur. J. Med. Chem..

[cit20] Fisher J. F., Shahriar M. (2006). Recent advances in MMP inhibitor design. Cancer Metastasis Rev..

[cit21] Hajri M., Esteve M., Khoumeri O., Abderrahim R., Montana T. M., Vanelle P. (2016). Synthesis and evaluation of *in vitro* antiproliferative activity of new ethyl 3-(arylethynyl)quinoxaline-2-carboxylate and pyrido[4,3-*b*]quinoxalin-1(2*H*)-one derivatives. Eur. J. Med. Chem..

[cit22] Ingle R., Marathe R., Magar D., Patel H. M., Surana S. J. (2013). Sulphonamidoquinoxalines: search for anticancer agent. Eur. J. Med. Chem..

[cit23] Piras S., Loriga M., Paglietti G. (2004). Quinoxaline chemistry. Part XVII. Methyl 4-(substituted 2-quinoxalinyloxy) phenyl acetates and ethyl *N*-(4-(substituted 2-quinoxalinyloxy) phenyl acetyl) glutamates analogs of methotrexate: synthesis and evaluation of *in vitro* anticancer activity. Farmaco.

[cit24] Khan S. A., Saleem K., Khan Z. (2007). Synthesis, characterization an *in vitro* antibacterial activity of new steroidal thiazolo quinoxalines. Eur. J. Med. Chem..

[cit25] Ramalingam P., Ganapaty S., Rao Ch. B. (2010). *In vitro* antitubercular and antimicrobial activities of 1-substituted quinoxaline-2,3(1*H*,4*H*)-diones. Bioorg. Med. Chem. Lett..

[cit26] Burguete A., Pontiki E., Hadjipavlou-Litina D., Ancizu S., Villar R. (2011). *et al.*, Synthesis and Biological evaluation of New Quinoxaline Derivatives as Antioxidant and Anti-Inflammatory Agents. Chem. Biol. Drug Des..

[cit27] Abu-Hashem A. A., Gouda M. A., Badria F. A. (2010). Synthesis of some new pyrimido [2′,1′:2,3]thiazolo[4,5-*b*]quinoxaline derivatives as anti-inflammatory and analgesic agents. Eur. J. Med. Chem..

[cit28] Fathalla W. (2015). Chemoselective synthesis of 3,6,7-trisubstituted 2-(2,3:5,6-di-*O*-isopropylidene-β-d-mannofuranosyloxy)- and 2-(2-acetamido-3,4,6-tri-*O*-acetyl-2-deoxy-β-d-glucopyranosyloxy)quinoxaline derivatives. Chem. Heterocycl. Compd..

[cit29] El Rayes S. M. (2008). Convenient synthesis of some methyl-*N*-[2-(3-oxo-6-*p*-tolyl-2,3,4,5-tetrahydropyridazin-2-yl)-acetylamino]amino acid esters. ARKIVOC.

[cit30] Megahed M., Fathalla W., Elsheikh A. (2018). Synthesis and antimicrobial activity of methyl (2-(2-(2-arylquinazolin-4-yl)sulfanyl)acetyl)amino alkanoates. J. Heterocycl. Chem..

[cit31] El Rayes S. M., Ali I. A. I., Fathalla W., Mahmoud M. A. (2020). Synthesis and biological activities of some new benzotriazinone derivatives based on molecular docking; promising HepG2 liver carcinoma inhibitors. ACS Omega.

[cit32] Aboelmagd A., Alotaibi S. H., El Rayes S. M., Elsayed G. M., Ali I. A. I., Fathalla W., Pottoo F. H., Khan F. A. (2020). Synthesis and Anti proliferative Activity of New *N-*Pentylquinoxaline carboxamides and Their *O*-Regioisomer. ChemistrySelect.

[cit33] Aboelmagd A., El Rayes S. M., Gomaa M. S., Ali I. A. I., Fathalla W., Pottoo F. H., Khan F. A., Khalifa M. E. (2021). The synthesis and antiproliferative activity of new *N*-allyl quinoxalinecarboxamides and their *O*–regioisomers. New J. Chem..

[cit34] Abad N., Sallam H. H., Al-Ostoot H. F., Khamees H. A., Al-horaibi S. A., Sridhar M. A., Shaukath A. K., Mahendra M., El Hafi M., Mague J. T., El Mokhtar E., Ramli Y. (2021). Synthesis, crystal structure, DFT calculations, Hirshfeld surface analysis, energy frameworks, molecular dynamics and docking studies of novel isoxazolequinoxaline derivative. J. Mol. Struct..

[cit35] Kánai K., Arányi P., Böcskei Z., Ferenczy G., Harmat V., Simon K., Bátori S., Náray-Szabo G., Hermecz I. (2008). Prolyl oligopeptidase inhibition by *N*-acyl-pro-pyrrolidine-type molecules. J. Med. Chem..

[cit36] Rajachandrasekhar V., Hariprasad C., Rao V. V., Venkataiah S., Dubey P. K. (2014). Synthesis of 9,10-Substituted 3,4,10,10*a*-Tetrahydro-2*H*,9*H*-1-oxa-4*a*,9-diazaphenanthrenes by Reductive Cyclization Method. Asian J. Chem..

[cit37] Druey J., Hüni A. (1952). Heilmittelchemische Studien in der heterocyclischen Reihe. III. Mitteilung. Quaternäre Verbindungen der Chinoxalinreihe. Helv. Chim. Acta.

[cit38] Núñez-Rico J. L., Vidal-Ferran A. (2013). [Ir(P–OP)]-Catalyzed Asymmetric Hydrogenation of Diversely Substituted C=N-Containing Heterocycles. Org. Lett..

[cit39] Son J. H., Zhu J. S., Phuan P. W., Cil O., Teuthorn A. P., Ku C. K., Lee S., Verkman A. S., Kurth M. J. (2017). High-Potency Phenylquinoxalinone Cystic Fibrosis Transmembrane Conductance Regulator (CFTR) Activators. J. Med. Chem..

[cit40] Ali I. A. I., Al-Masoudi I. A., Hassan H. Gh., Al-Masoudi N. A. (2007). Synthesis and anti-HIV activity of new homo acyclic nucleosides, 1-(pent-4-enyl)quinoxalin-2-ones and 2-(pent-4-enyloxy)quinoxalines. Chem. Heterocycl. Compds.

[cit41] El-Hawash S. A. M., Habib N. S., Kassem M. A. (2006). Synthesis of some new quinoxalines and 1,2,4-triazolo[4,3-*a*]-quinoxalines for evaluation of *in vitro* antitumor and antimicrobial activities. Arch. Pharm..

[cit42] Xue Z.-Y., Jiang Y., Peng X.-Z., Yuan W.-C., Zhang X.-M. (2010). The First General, Highly Enantioselective Lewis Base Organo-catalyzed Hydrosilylation of Benzoxazinones and Quinoxalinones. Adv. Synthesis and Catalysis.

[cit43] Zhongrong S., Chunle W., Sikai W., Wenbo Z., Runjiang S., Deyu H. (2022). Synthesis, Anti-Potato Virus Y Activities, and Interaction Mechanisms of Novel Quinoxaline Derivatives Bearing Dithioacetal Moiety. J. Agric. Food Chem..

[cit44] Ali I. A. I., El Rayes S. M. (2014). Synthesis of quinoxaline reverse ribofuranosides and their *O*-regioisomers. Monatshefte fur Chemie.

[cit45] Fathalla W., Ali I. A. I., Pazdera P. (2017). A novel method for heterocyclic amide–thioamide transformations. Beilstein J. Org. Chem..

[cit46] Rao K. R., Raghunadh A., Mekala R., Meruva S. B., Ganesh K. R., Krishna T., Kalita D., Laxminarayana E., Pal M. (2016). Synthesis of Novel Drug-Like Small Molecules Based on Quinoxaline Containing Amino Substitution at C-2. J. Heterocycl. Chem..

[cit47] Magnus R., Francisco T., Fenia R. S. (2010). The First General, Efficient and Highly Enantioselective Reduction of Quinoxalines and Quinoxalinones. Chem.–Eur. J..

[cit48] Han S.-Y., Kim Y.-A. (2004). Recent Development of Peptide Coupling Reagents in Organic Synthesis. Tetrahedron.

[cit49] Alahmari F., Rehman S., Almessiere M., Khan F. A., Slimani Y., Baykal A. (2021). Synthesis of Ni_0.5_Co_0.5−*x*_Cd_*x*_Fe_1.78_Nd_0.02_O_4_ (*x* ≤ 0.25) nanofibers by using electrospinning technique induce anti-cancer and anti-bacterial activities. J. Biomol. Struct. Dyn..

[cit50] Holzhauer L., Liagre C., Fuhr O., Jung N., Bräse S. (2022). Scope of tetrazolo[1,5-*a*]quinoxalines in CuAAC reactions for the synthesis of triazoloquinoxalines, imidazoloquinoxalines, and rhenium complexes thereof. Beislstein J. Org. Chem..

[cit51] Rose P. W., Prlic A., Altunkaya A., Bi C., Bradley A. R., Christie C. H., Costanzo L. D., Duarte J. M., Dutta S., Feng Z., Green R. K., Goodsell D. S., Hudson B., Kalro T., Lowe R., Peisach E., Randle C., Rose A. S., Shao C., Tao Y. P., Valasatava Y., Voigt M., Westbrook J. D., Woo J., Yang H., Young J. Y., Zardecki C., Berman H. M., Burley S. K. (2017). The RCSB protein data bank: integrative view of protein, gene and 3D structural information. Nucleic Acids Res..

[cit52] Burley S. K., Bhikadiya C., Bi C., Bittrich S., Chen L., Crichlow G. V., Christie C. H., Dalenberg K., Di Costanzo L., Duarte J. M., Dutta S., Feng Z., Ganesan S., Goodsell D. S., Ghosh S., Green R. K., Guranovic V., Guzenko D., Hud-son B. P., Lawson C. L., Liang Y., Lowe R., Namkoong H., Peisach E., Persikova I., Randle C., Rose A., Rose Y., Sali A., Segura J., Sekharan M., Shao C., Tao Y. P., Voigt M., Westbrook J. D., Young J. Y., Zardecki C., Zhuravleva M. (2021). RCSB Protein Data Bank: powerful new tools for exploring 3D structures of biological macromolecules for basic and applied research and education in fundamental biology, biomedicine, biotechnology, bioengineering and energy sciences. Nucleic Acids Res..

[cit53] Lu C., Wu C., Ghoreishi D., Chen W., Wang L., Damm W., Ross G. A., Dahlgren M. K., Russell E., Von Bargen C. D., Abel R., Friesner R. A., Harder E. D. (2021). OPLS4: Improving Force Field Accuracy on Challenging Regimes of Chemical Space. J. Chem. Theory Comput..

[cit54] Alves M. J., Froufe H. J., Costa A. F., Santos A. F., Oliveira L. G., Osorio S. R., Abreu R. M., Pintado M., Ferreira I. C. (2014). Docking studies in target proteins involved in antibacterial action mechanisms: extending the knowledge on standard antibiotics to antimicrobial mushroom compounds. Molecules.

[cit55] Kwofie S. K., Dankwa B., Odame E. A., Agamah F. E., Doe L. P. A., Teye J., Agyapong O., Miller 3rd W. A., Mosi L., Wilson M. D. (2018). In Silico Screening of Isocitrate Lyase for Novel Anti-Buruli Ulcer Natural Products Originating from Af-rica. Molecules.

[cit56] Jaundoo R., Bohmann J., Gutierrez G. E., Klimas N., Broderick G., Craddock T. J. A. (2018). Using a Consensus Docking Approach to Predict Adverse Drug Reactions in Combination Drug Therapies for Gulf War Illness. Int. J. Mol. Sci..

[cit57] El Rayes S. M., Aboelmagd A., Gomaa M. S., Ali I. A. I., Fathalla W., Pottoo F. H., Khan F. A. (2019). Convenient Synthesis and Anticancer Activity of Methyl 2-[3-(3-Phenyl-quinoxalin-2-ylsulfanyl)propanamido]alkanoates and *N*-Alkyl 3-((3-Phenyl-quinoxalin-2-yl)sulfanyl)propanamides. ACS Omega.

[cit58] Rehman S., Almessiere M. A., Khan F. A., Korkmaz A. D., Tashkandi N., Slimani Y., Baykal A. (2020). Synthesis and biological characterization of Mn_0.5_Zn_0.5_Eu_*x*_Dy_*x*_Fe_1.8−2*x*_O_4_ nanoparticles by sonochemical approach. Mater. Sci. Eng., C.

[cit59] Ansari M. A., Asiri S. M. M., Alzohairy M. A., Alomary M. N., Almatroudi A., Khan F. A. (2021). Biofabricated Fatty Acids-Capped Silver Nanoparticles as Potential Antibacterial, Antifungal, Antibiofilm and Anticancer Agents. Pharmaceuticals.

