# Supplementary material for: Synthesis and antiproliferative activity of 2-oxo-3-phenylquinoxaline derivatives and related compounds against colon cancer
Source: RSC Adv. 2024 Nov 8;14(48):35679–95. doi: 10.1039/d4ra06822j (PMC11544594; doi:10.1039/d4ra06822j)
Supplement: RA-014-D4RA06822J-s001 [file RA-014-D4RA06822J-s001.pdf]

This journal is © The Royal Society of Chemistry 2024

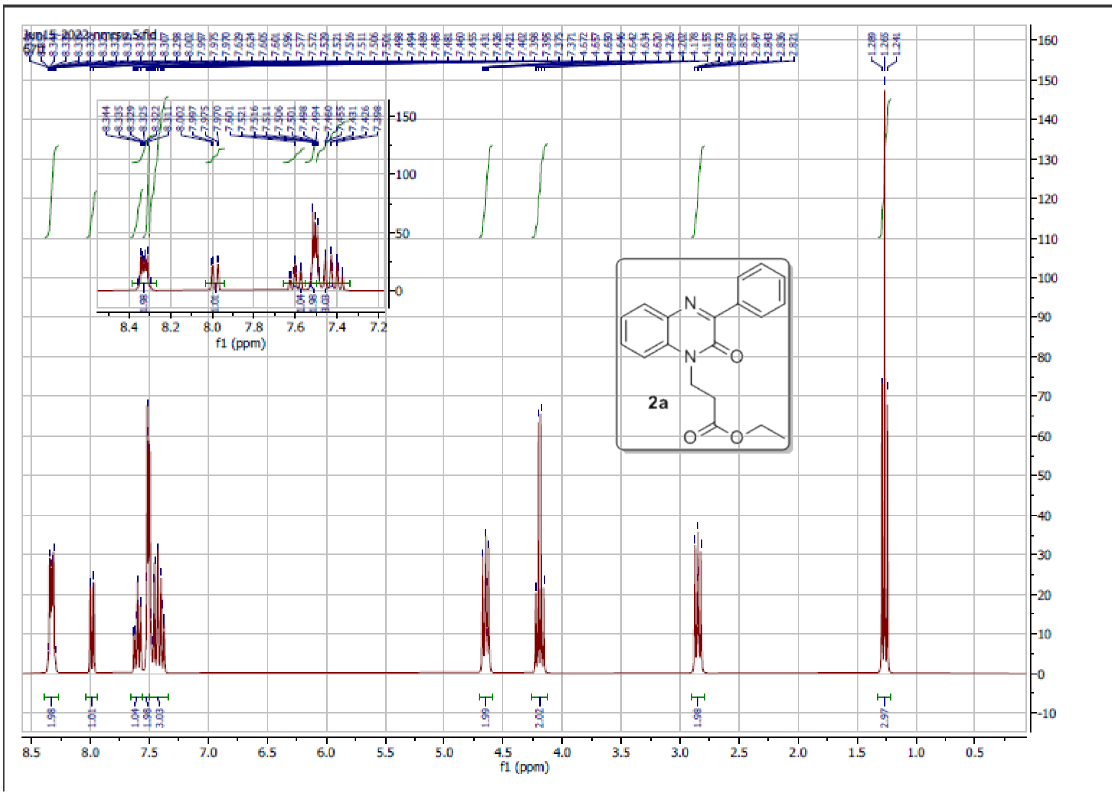

S1 ^{1}HNMR spectra of 2a

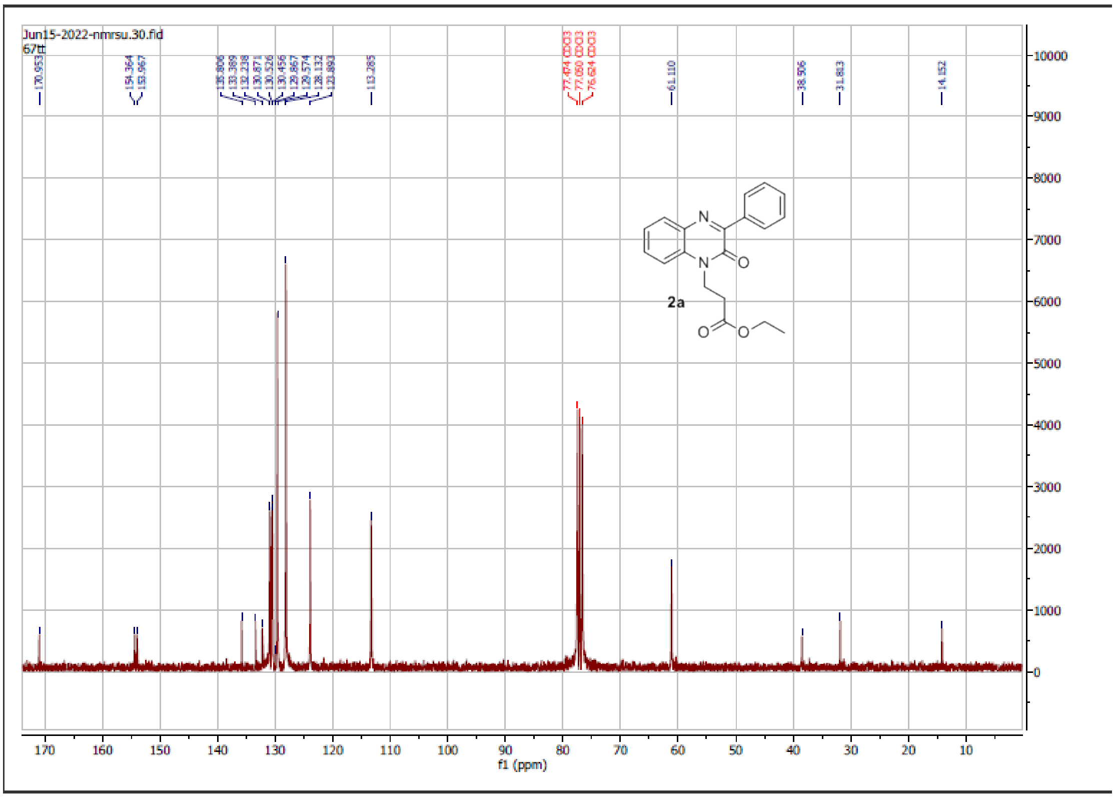

S2 ^{13}C NMR spectra of 2a

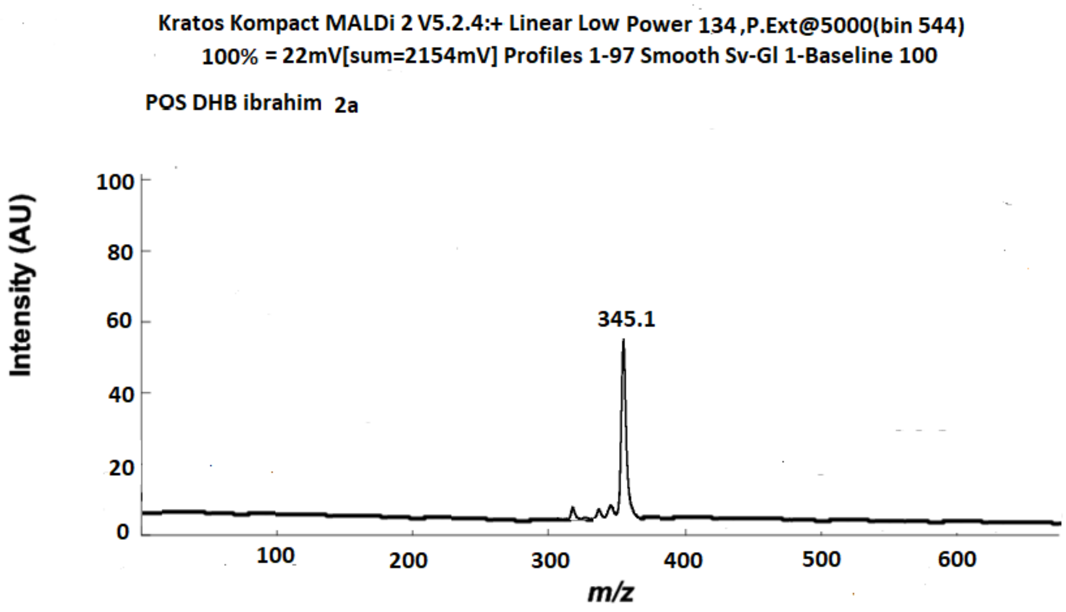

S3 MALDI spectra of 2a

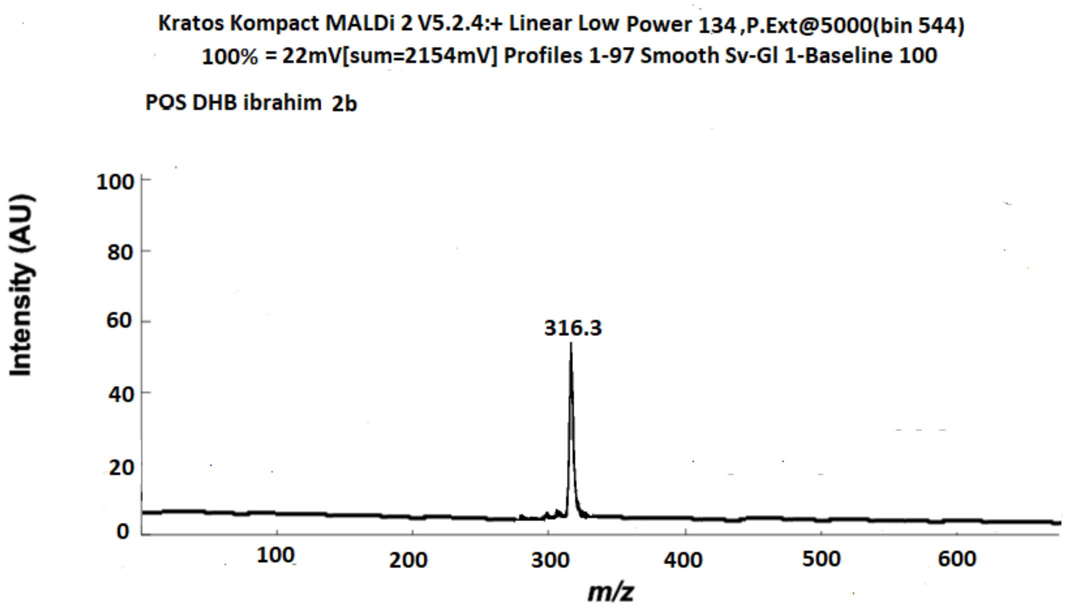

S4 MALDI spectra of 2b

Kratos Kompact MALDi 2 V5.2.4:+ Linear Low Power 134,P.Ext@5000(bin 544)
100% = 22mV[sum=2154mV] Profiles 1-97 Smooth Sv-Gl 1-Baseline 100

POS DHB ibrahim 2C

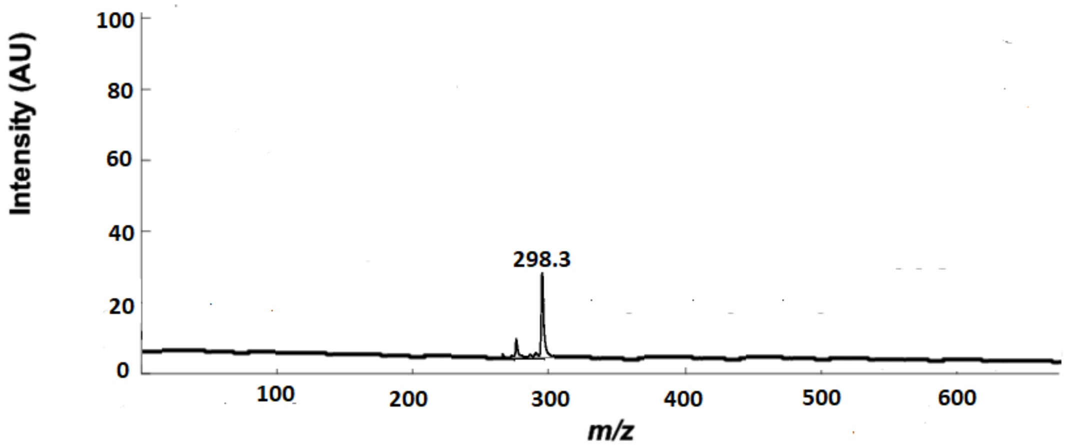

S5 MALDI spectra of 2c

Kratos Kompact MALDi 2 V5.2.4:+ Linear Low Power 134,P.Ext@5000(bin 544)
100% = 22mV[sum=2154mV] Profiles 1-97 Smooth Sv-Gl 1-Baseline 100

POS DHB ibrahim 3

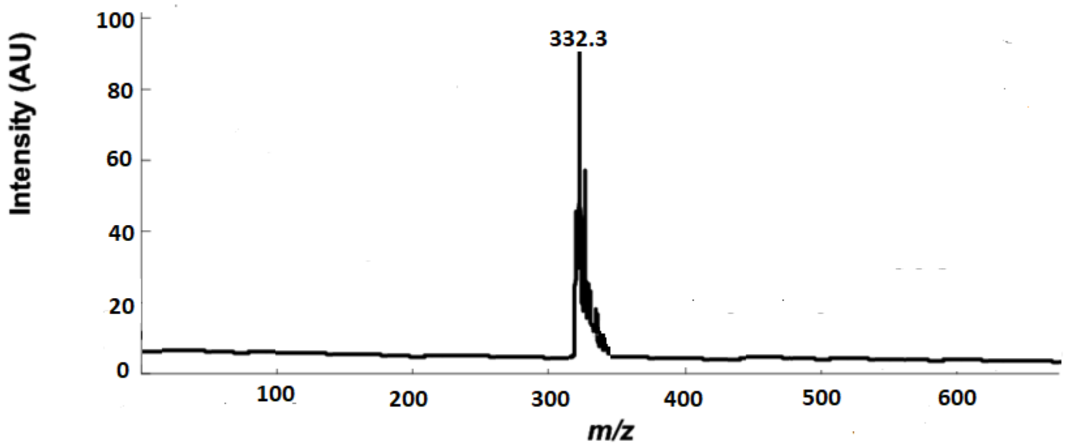

S6 MALDI spectra of 3

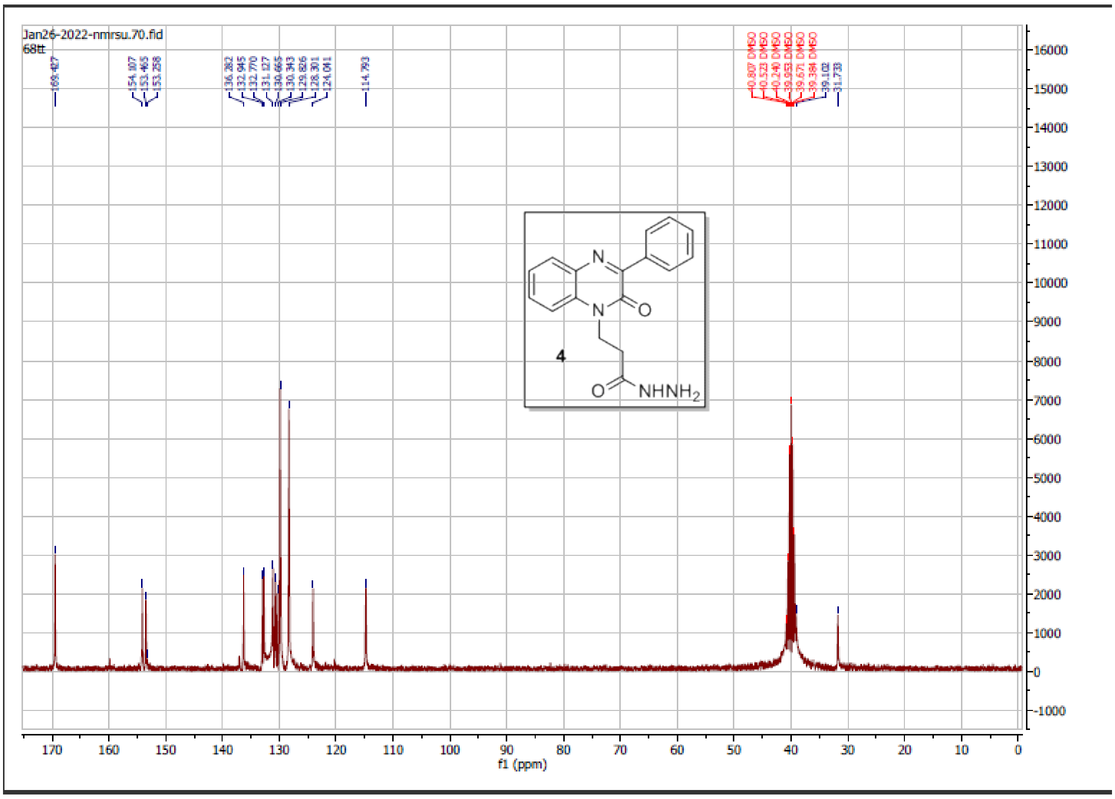

S7 ^{13}C NMR spectra of 4

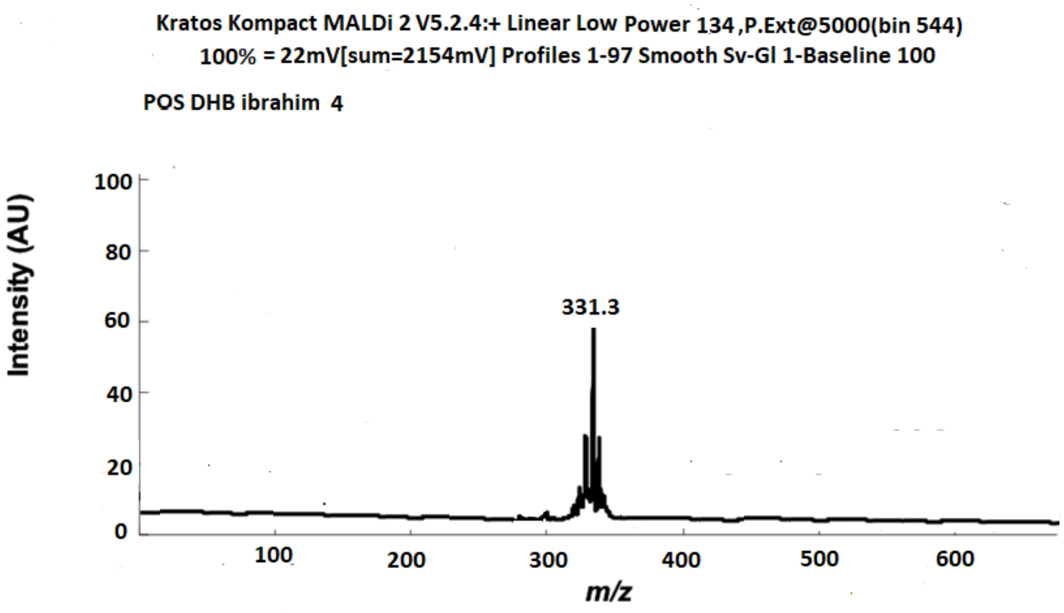

S8 MALDI spectra of 4

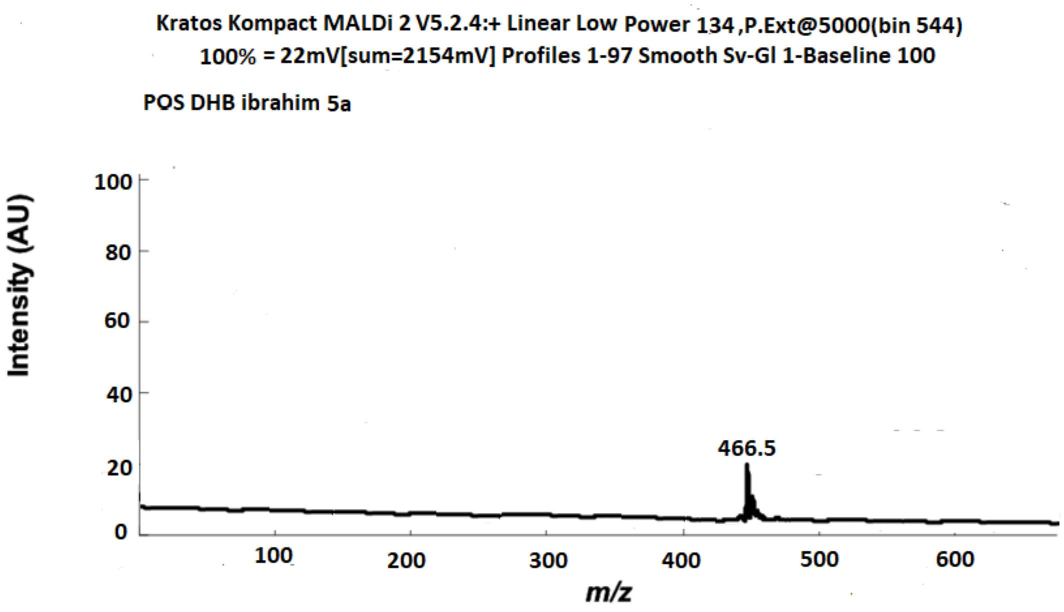

S9 MALDI spectra of 5a

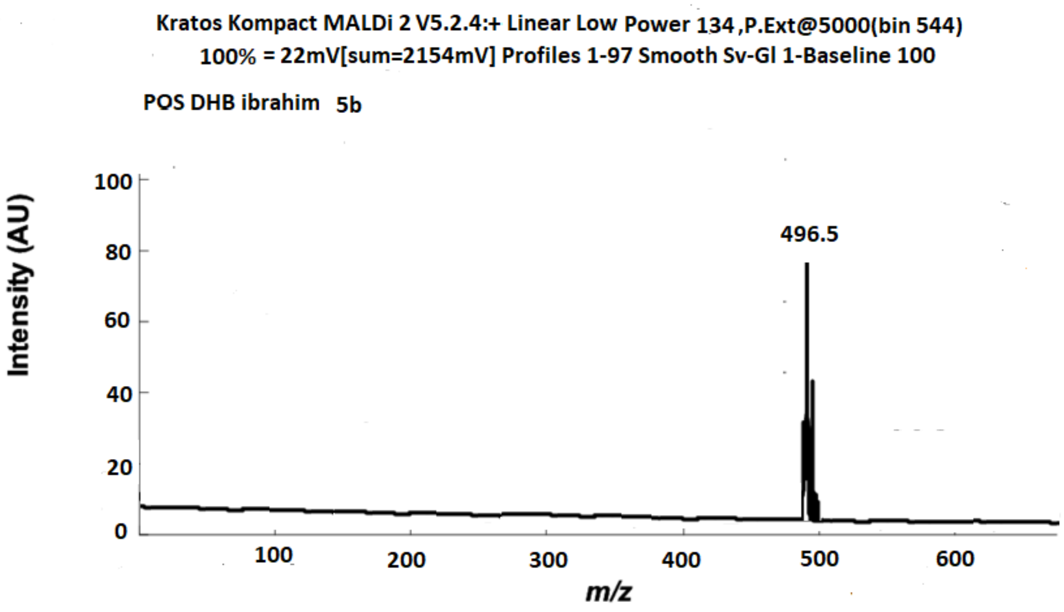

S10 MALDI spectra of 5b

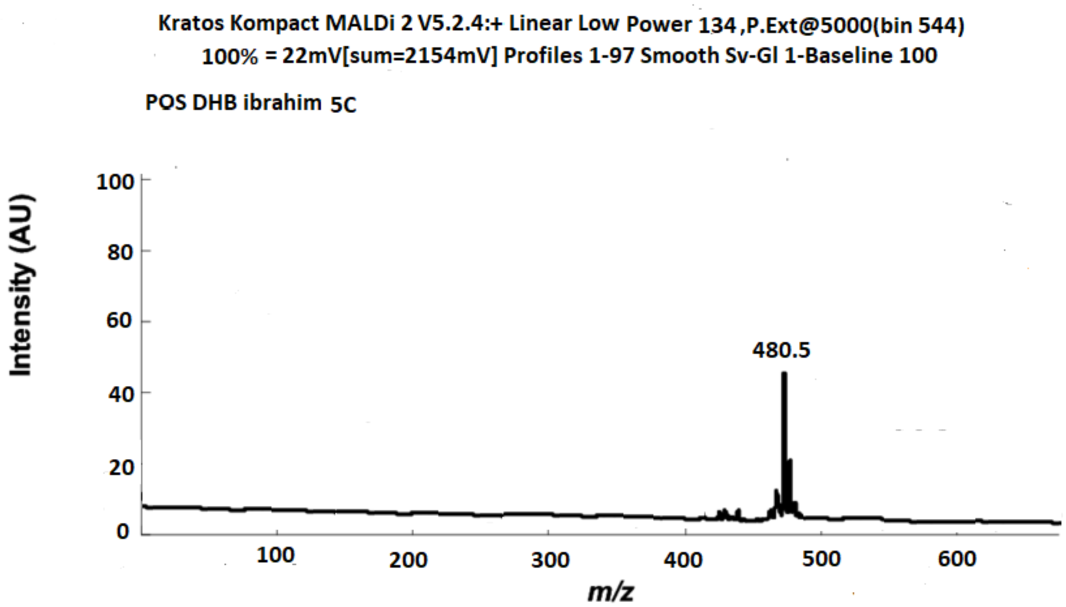

S11 MALDI spectra of 5c

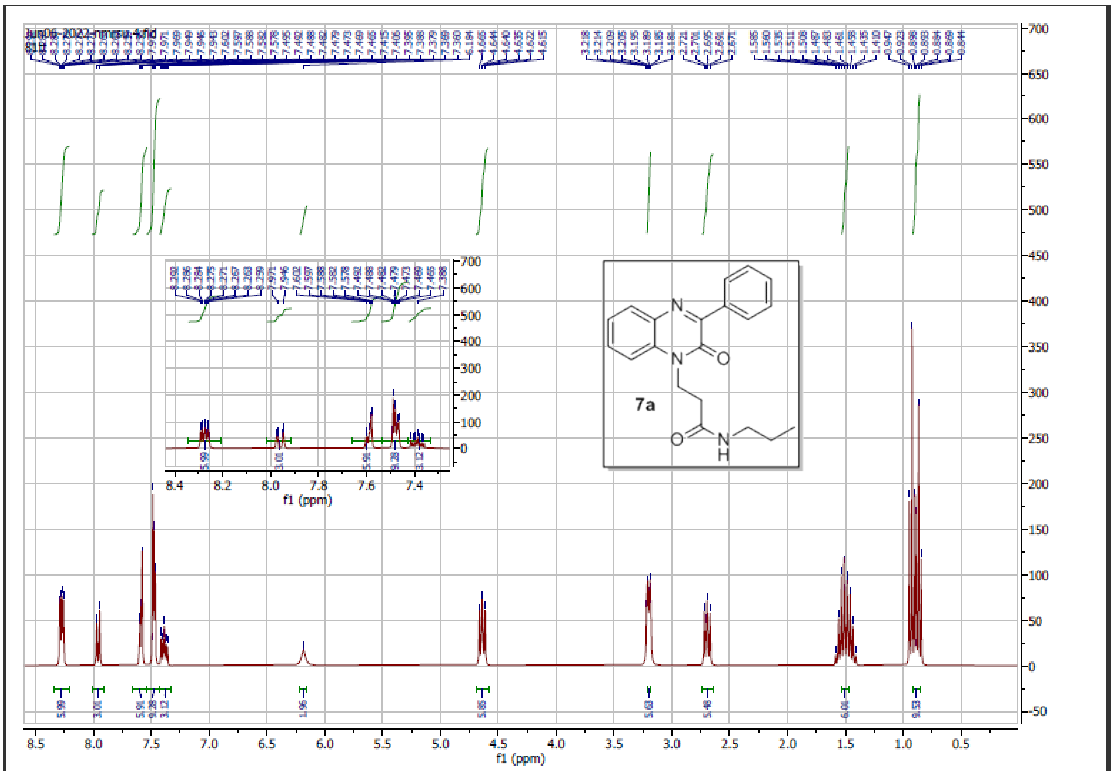

S12 ^{1}HNMR spectra of 7a

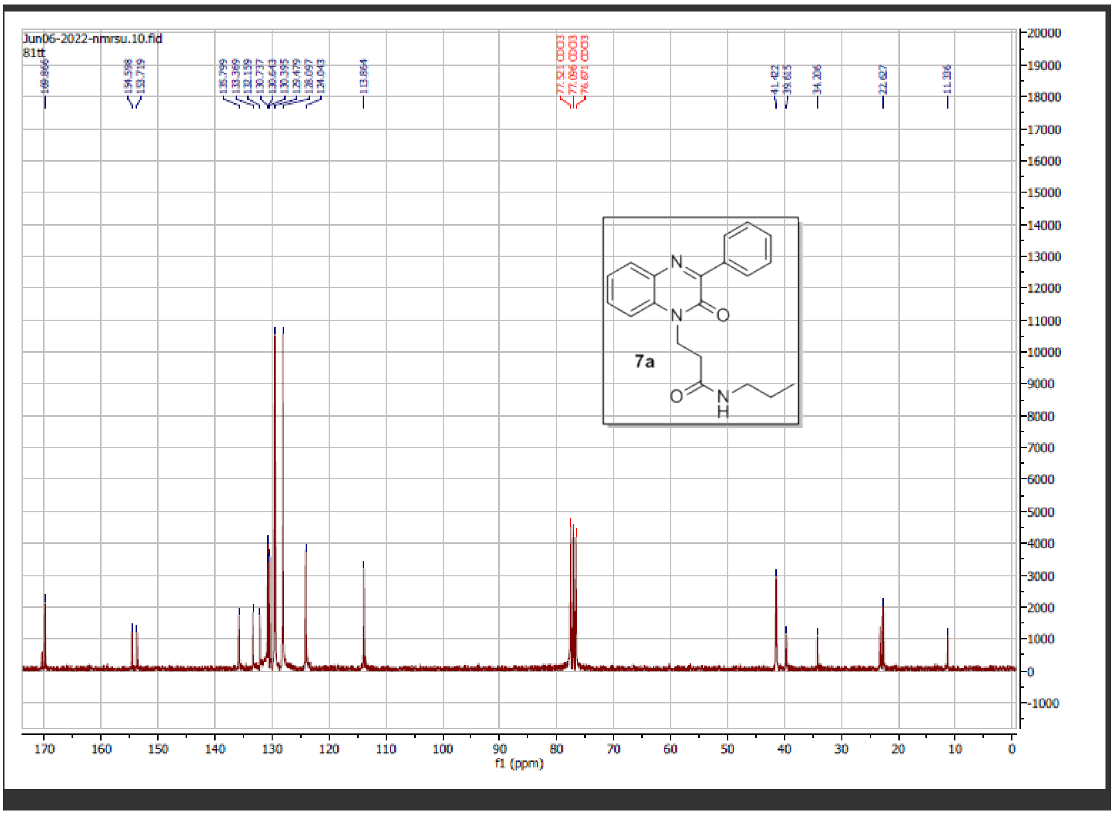

S13 ^{13}CNMR spectra of 7a

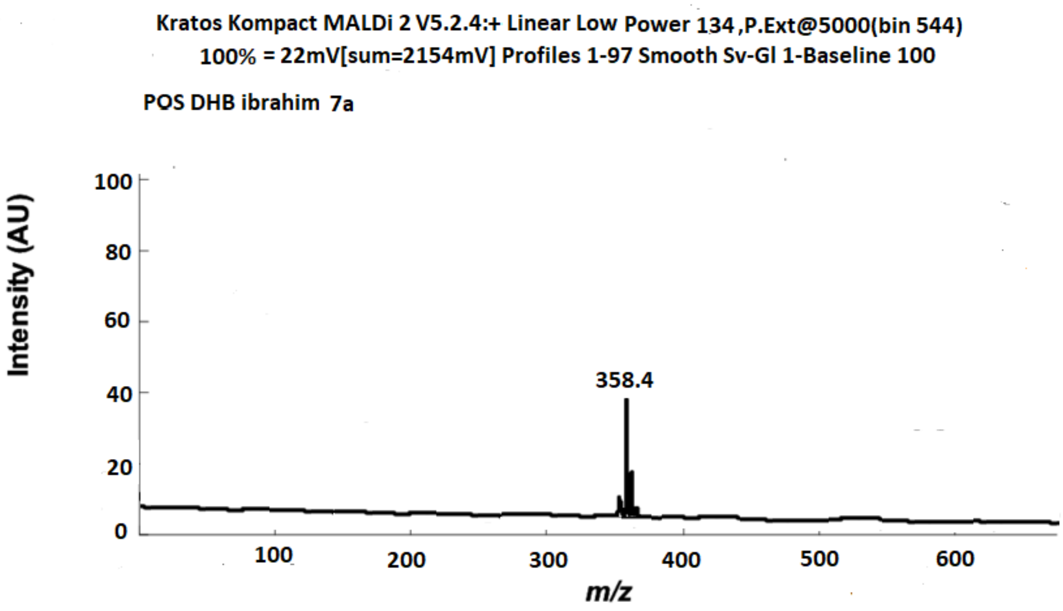

S14 MALDI spectra of 7a

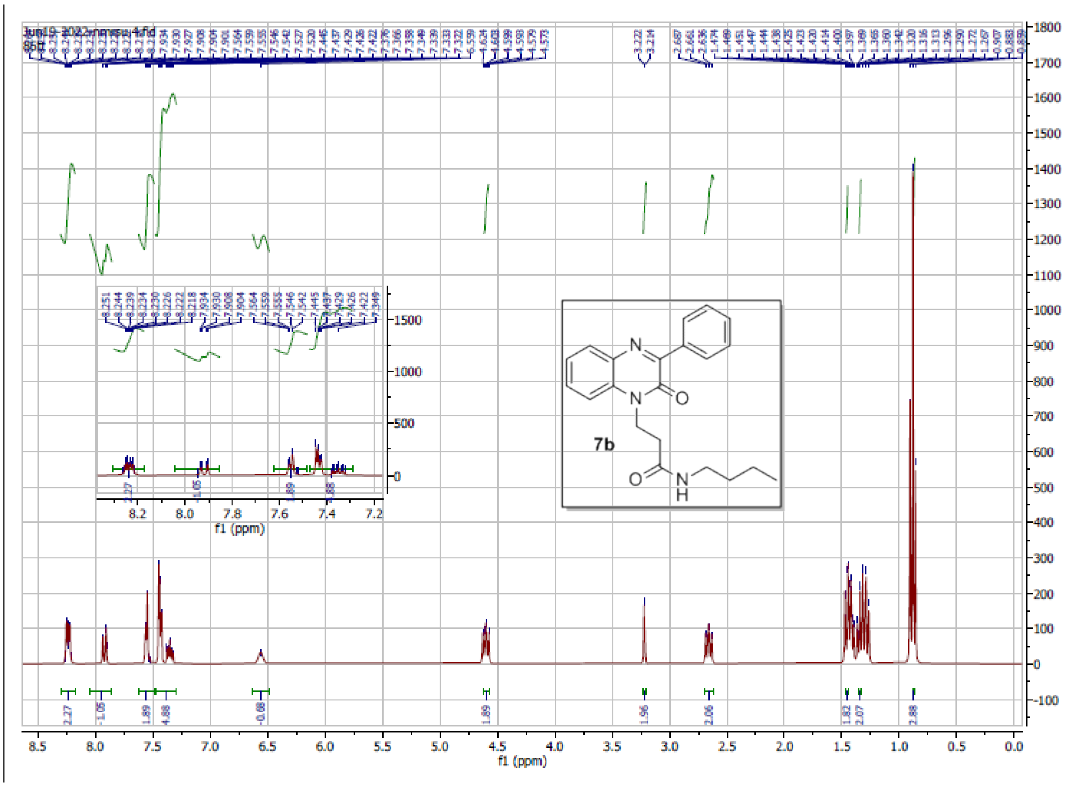

S15 ^{1}HNMR spectra of 7b

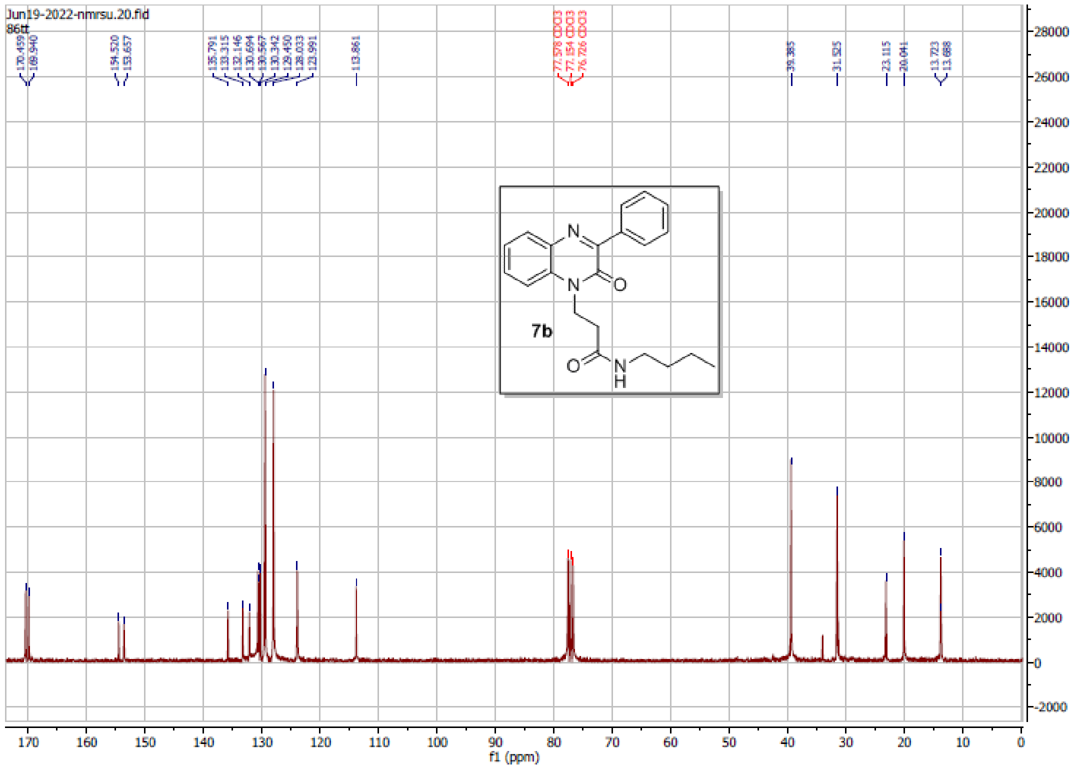

S16 ^{13}CNMR spectra of 7b

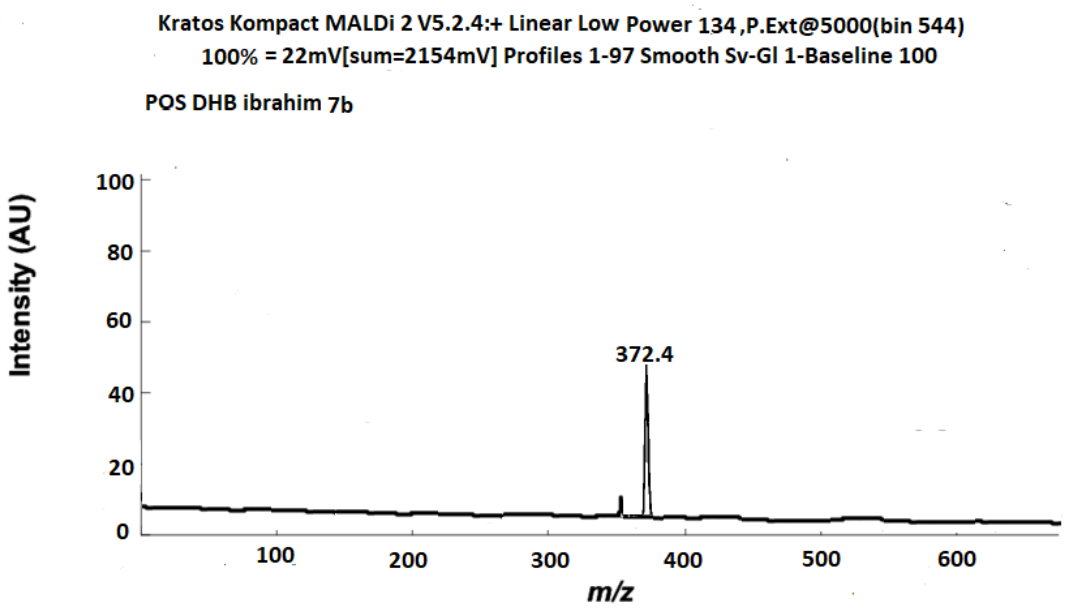

S17 MALDI spectra of 7b

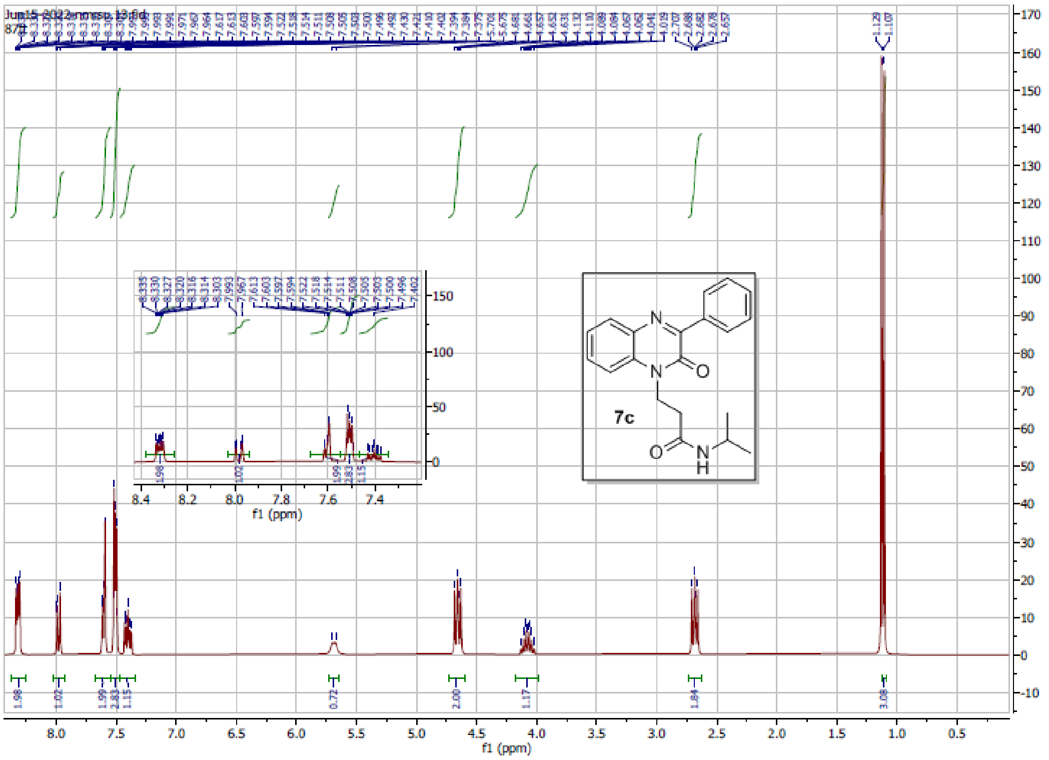

S18 ^{1}HNMR spectra of 7c

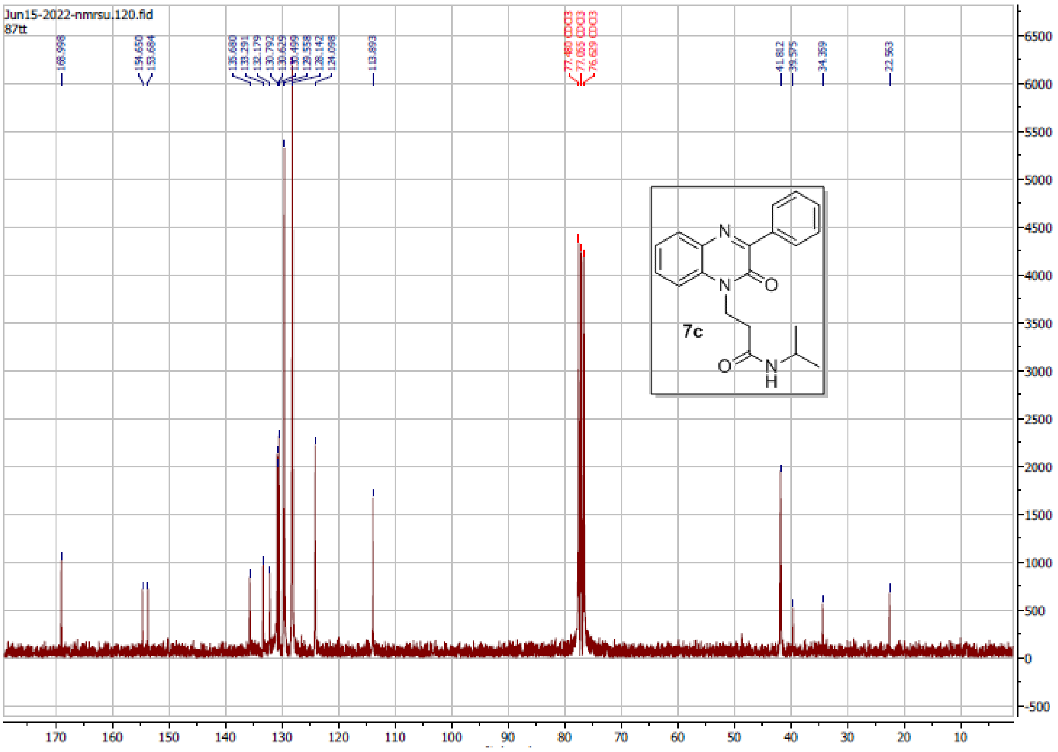

S19 ^{13}CNMR spectra of 7c

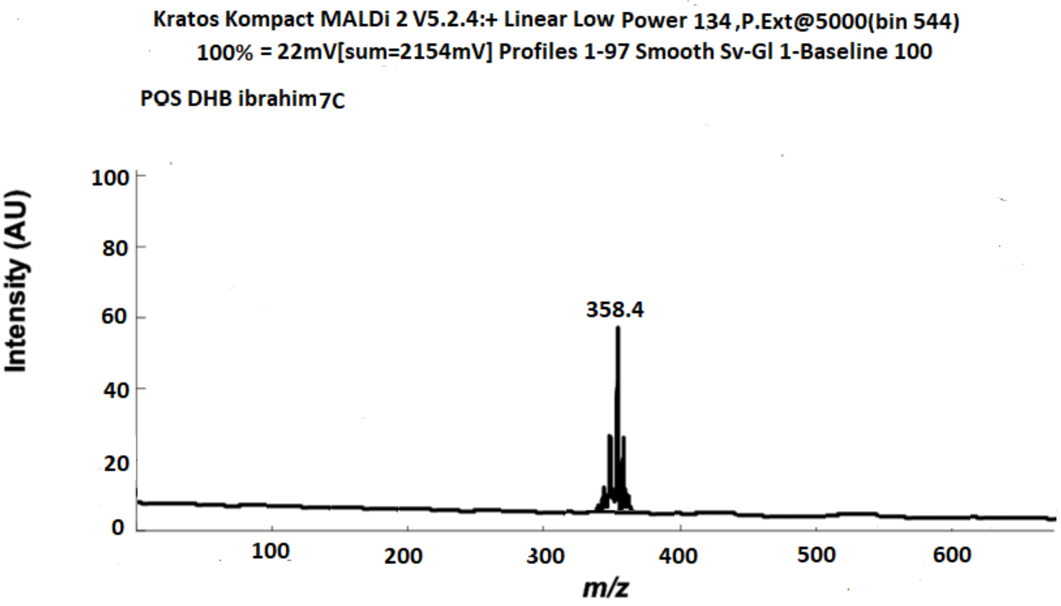

S20 MALDI spectra of 7c

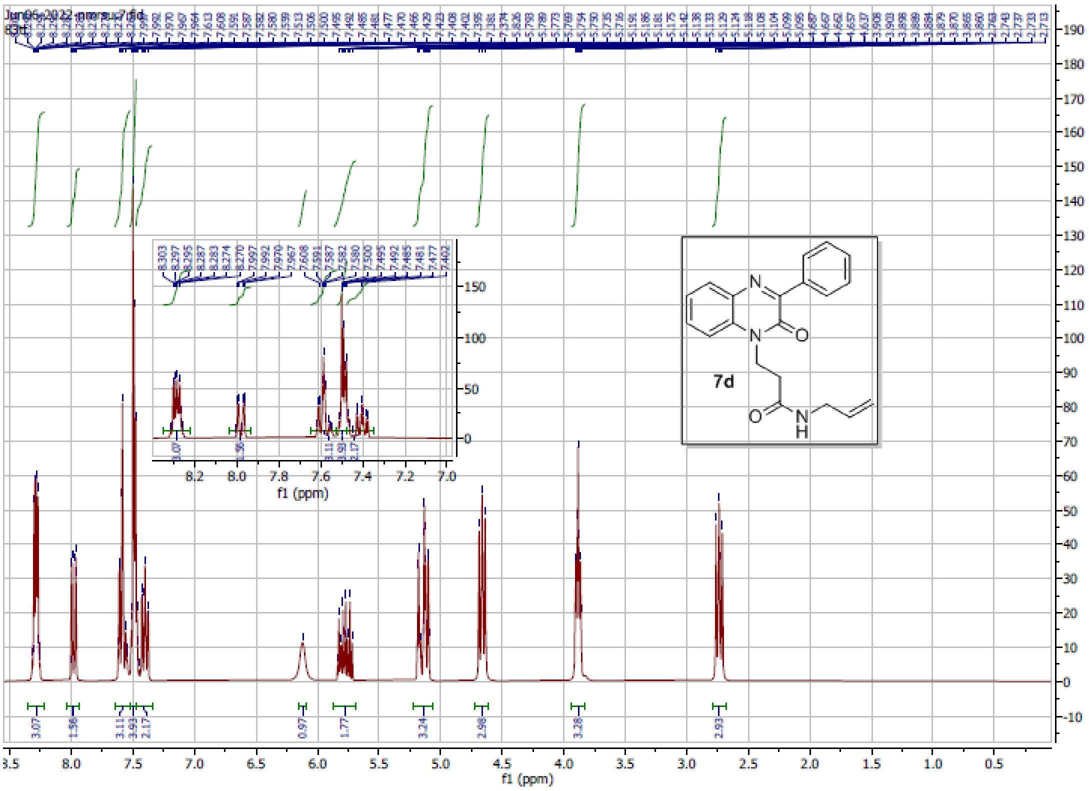

S21 ^{1}HNMR spectra of 7d

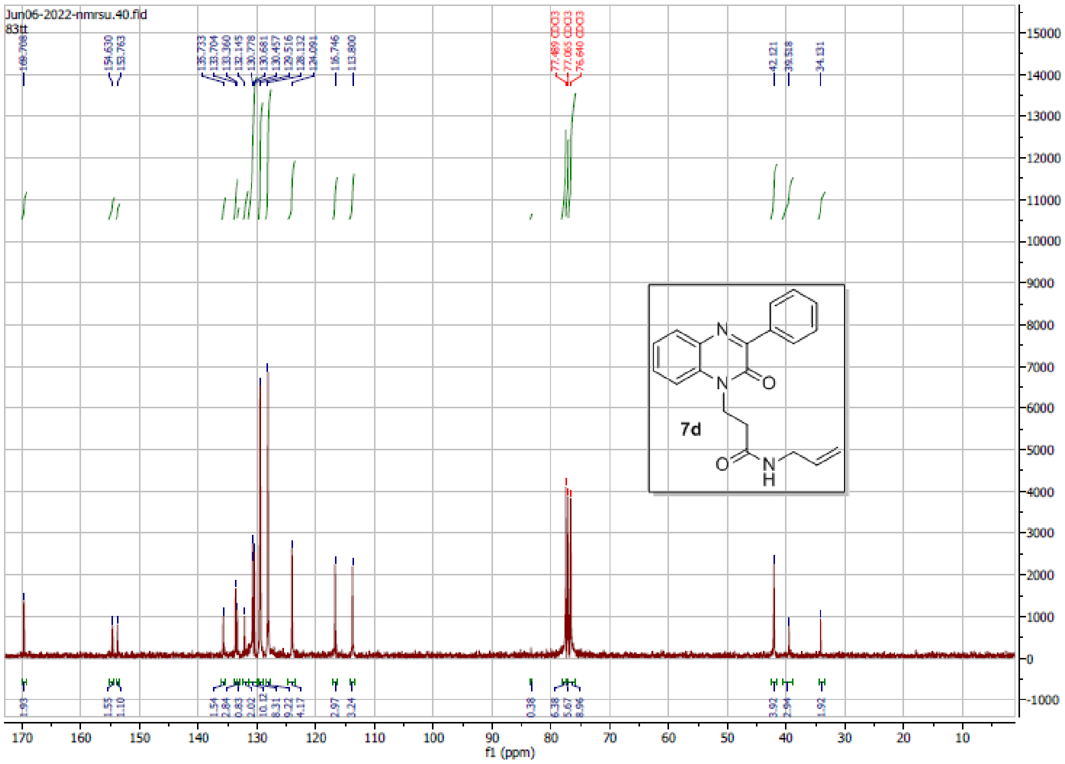

S22 ^{13}CNMR spectra of 7d

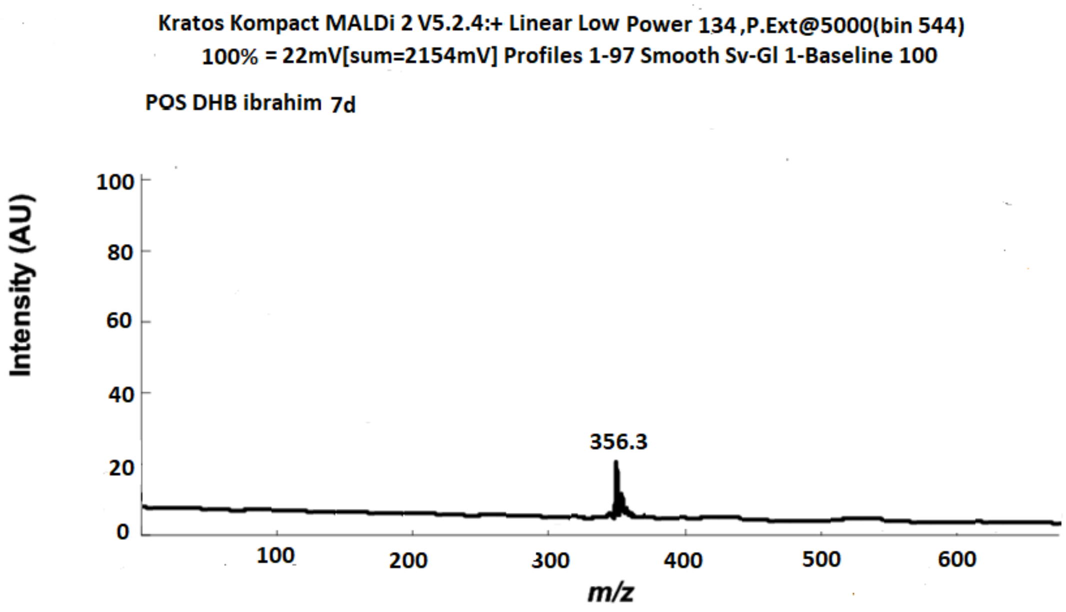

S23 MALDI spectra of 7d

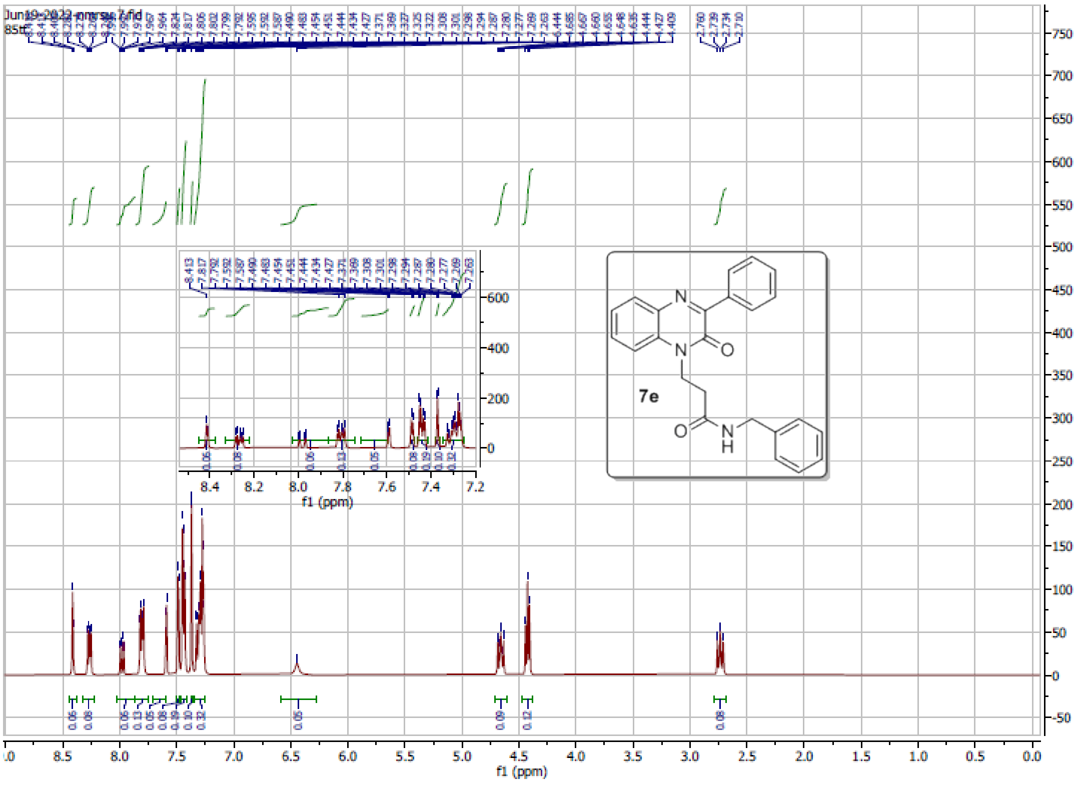

S24 ^{1}HNMR spectra of 7e

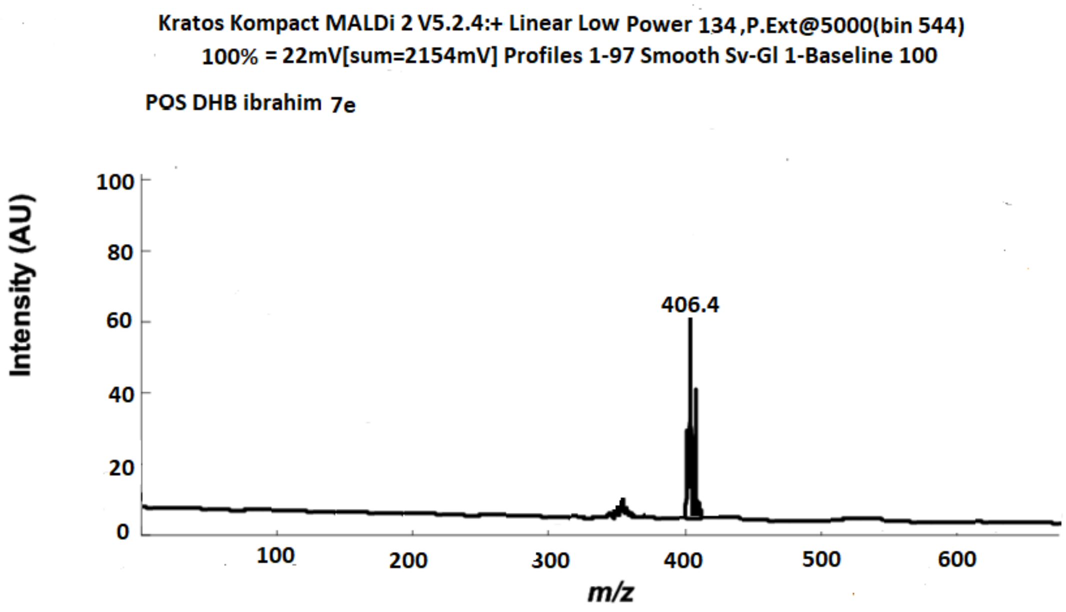

S25 MALDI spectra of 7e

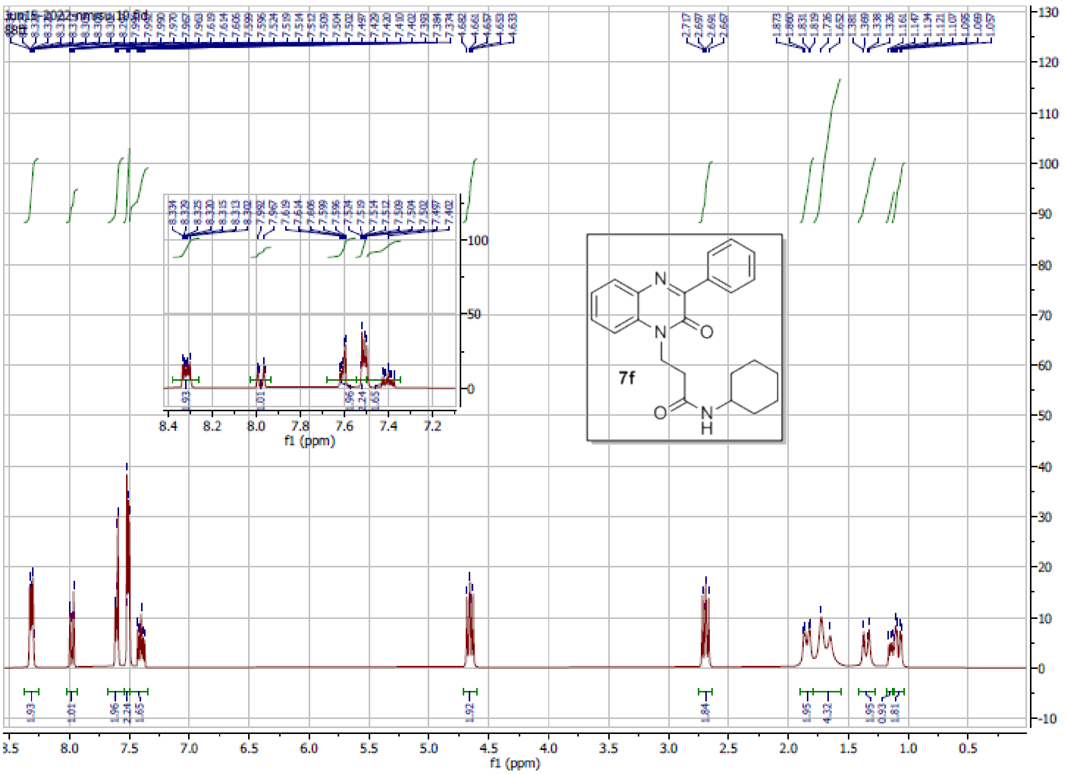

S26 ^{1}HNMR spectra of 7f

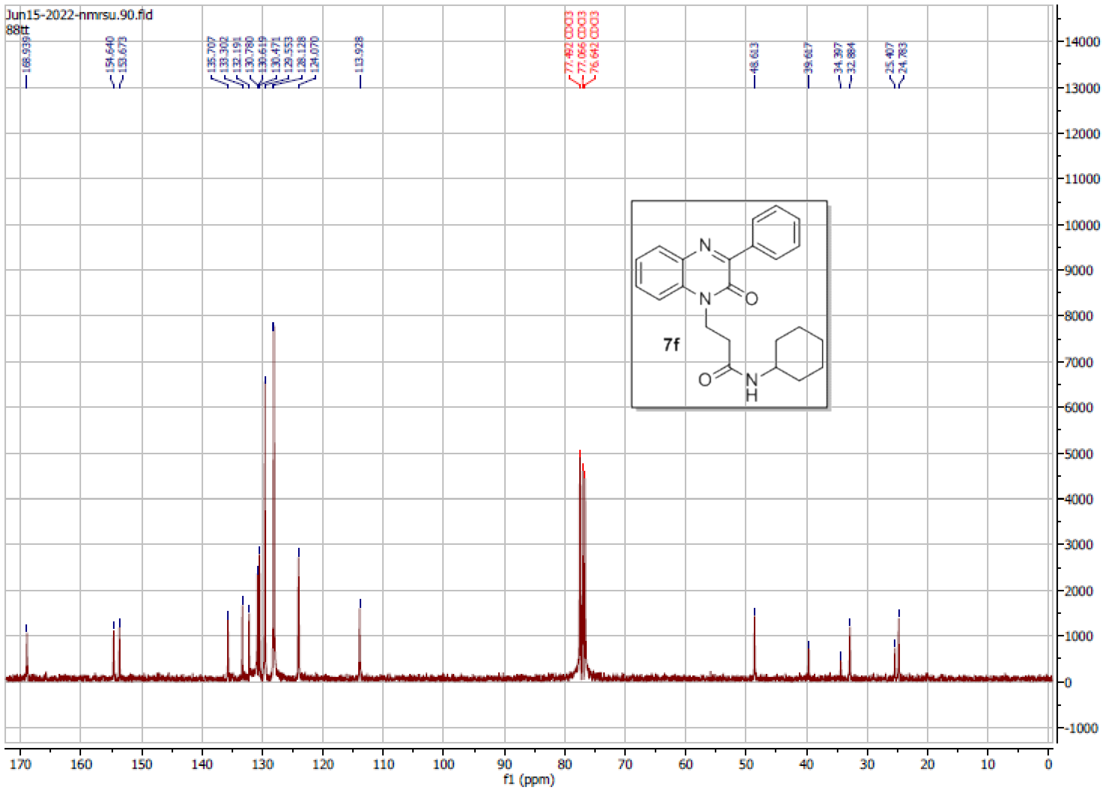

S27 ^{13}CNMR spectra of 7f

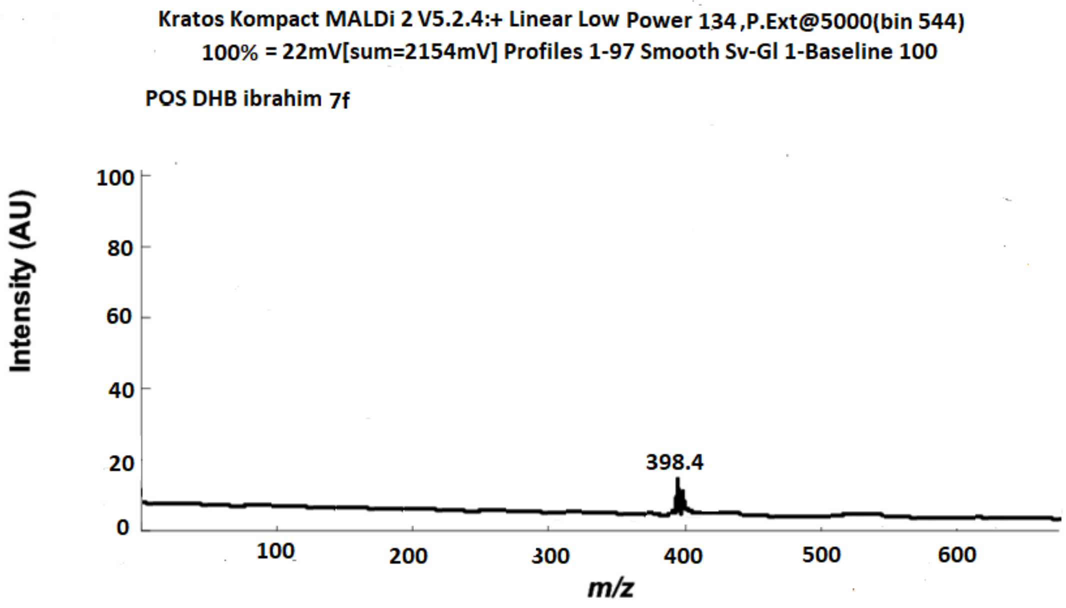

S28 MALDI spectra of 7f

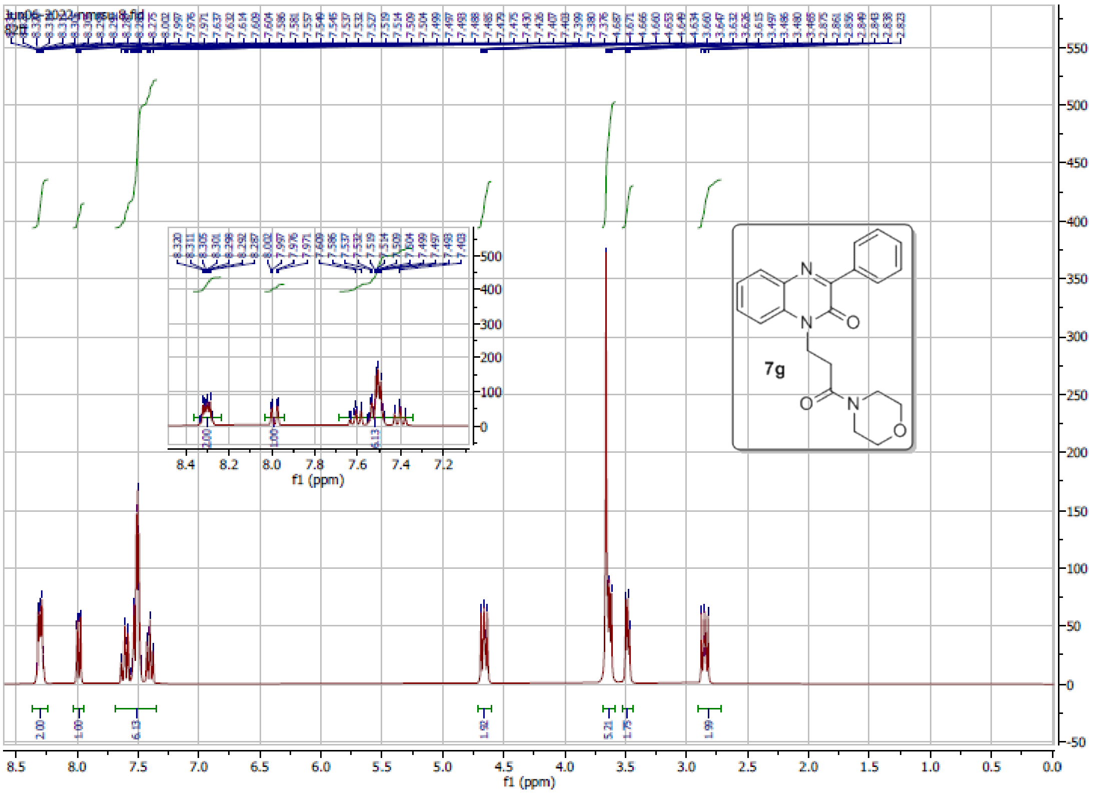

S29 ^{1}HNMR spectra of 7g

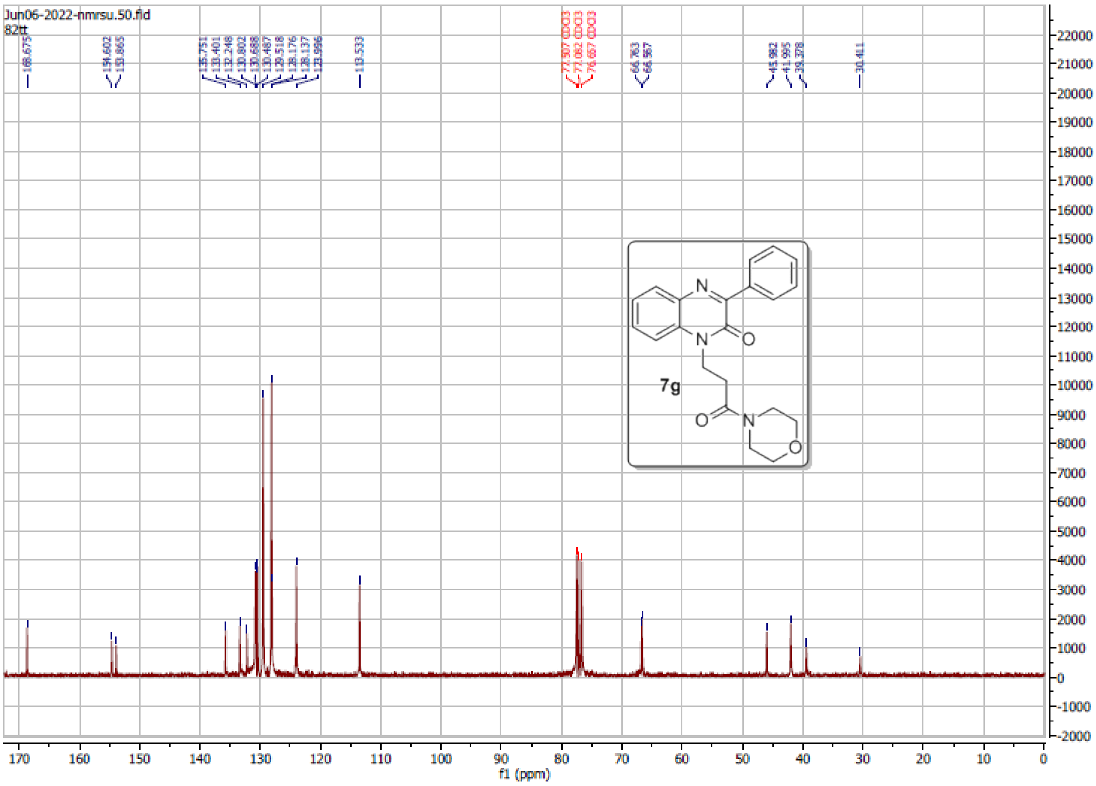

S30 ^{13}CNMR spectra of 7g

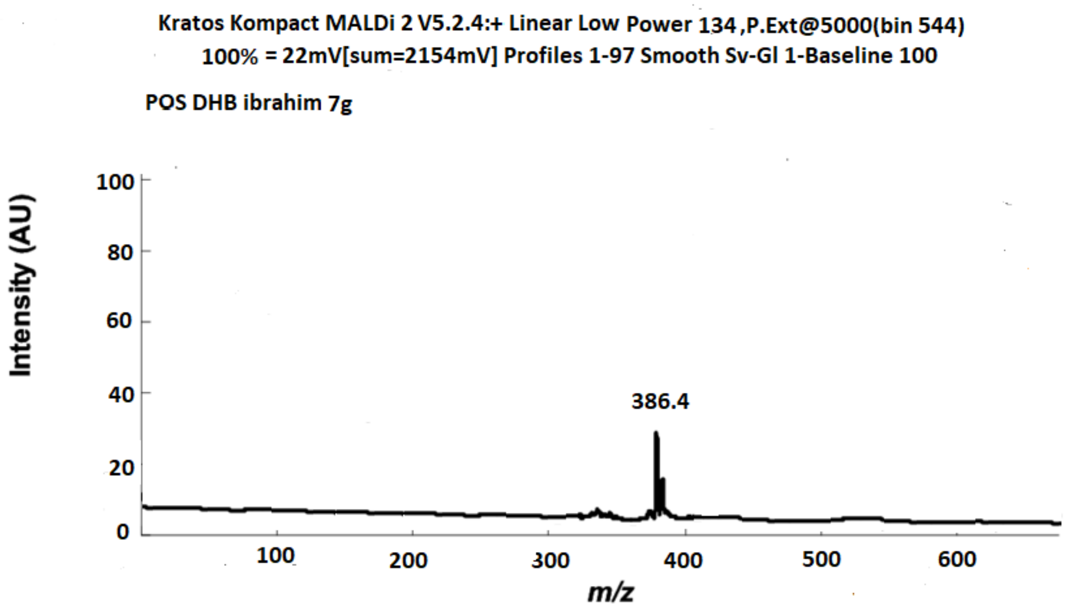

S31 MALDI spectra of 7g

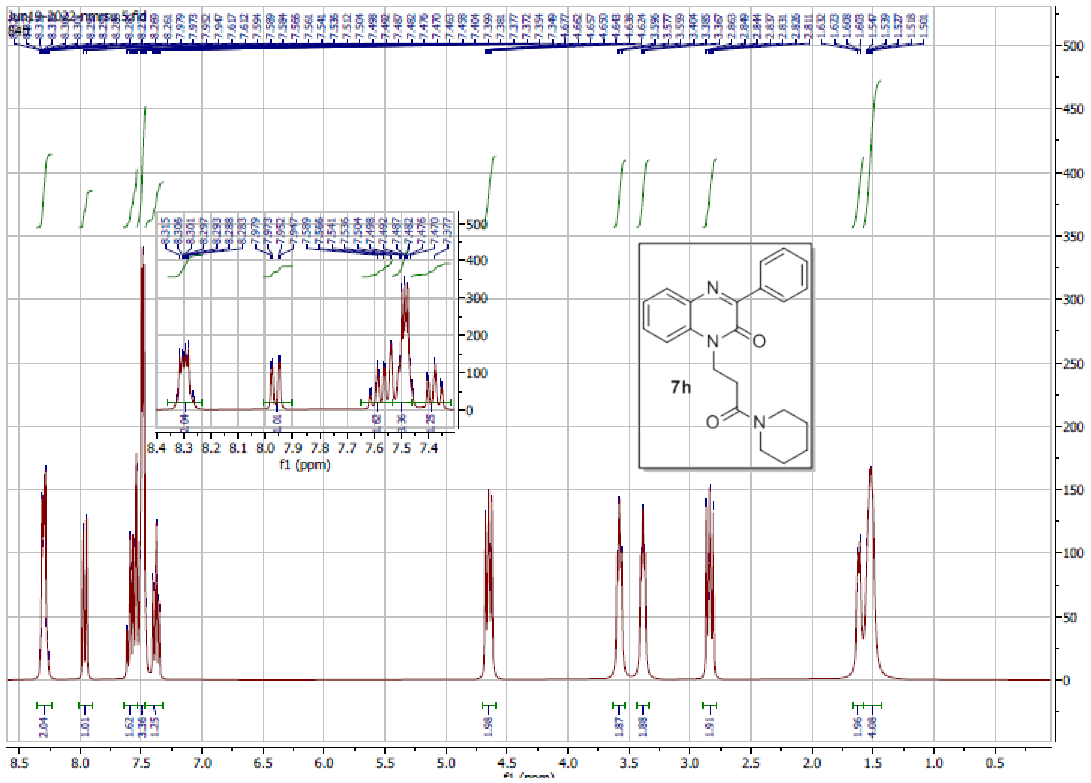

S32^{1}HNMR spectra of 7h

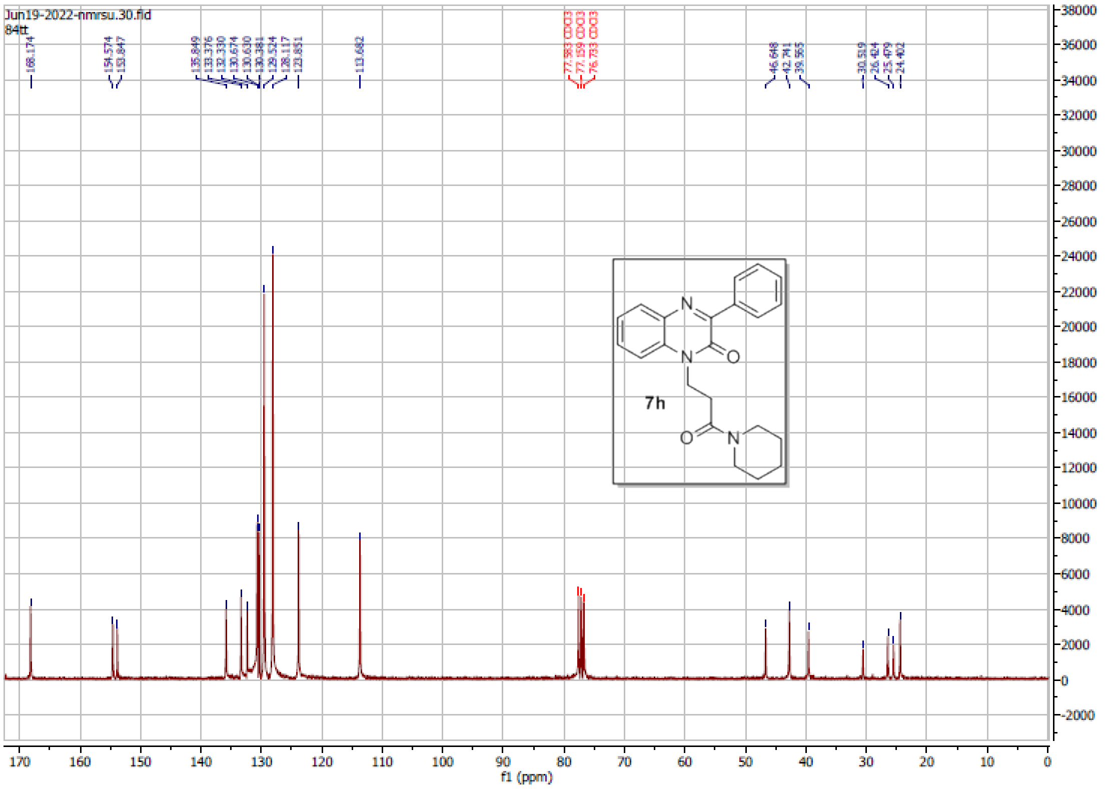

S33 ^{13}CNMR spectra of 7h

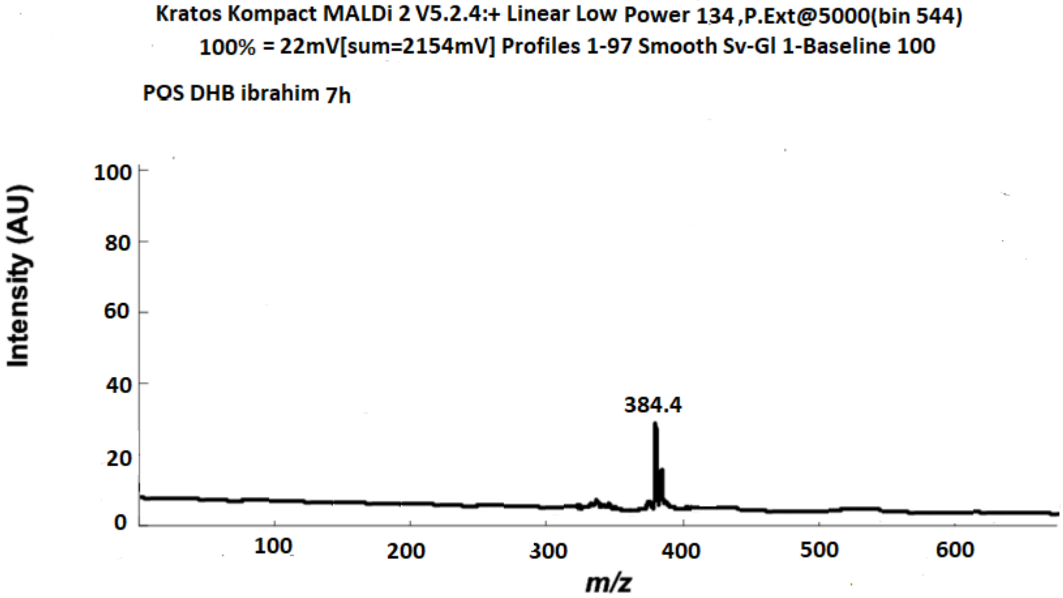

S34 MALDI spectra of 7h

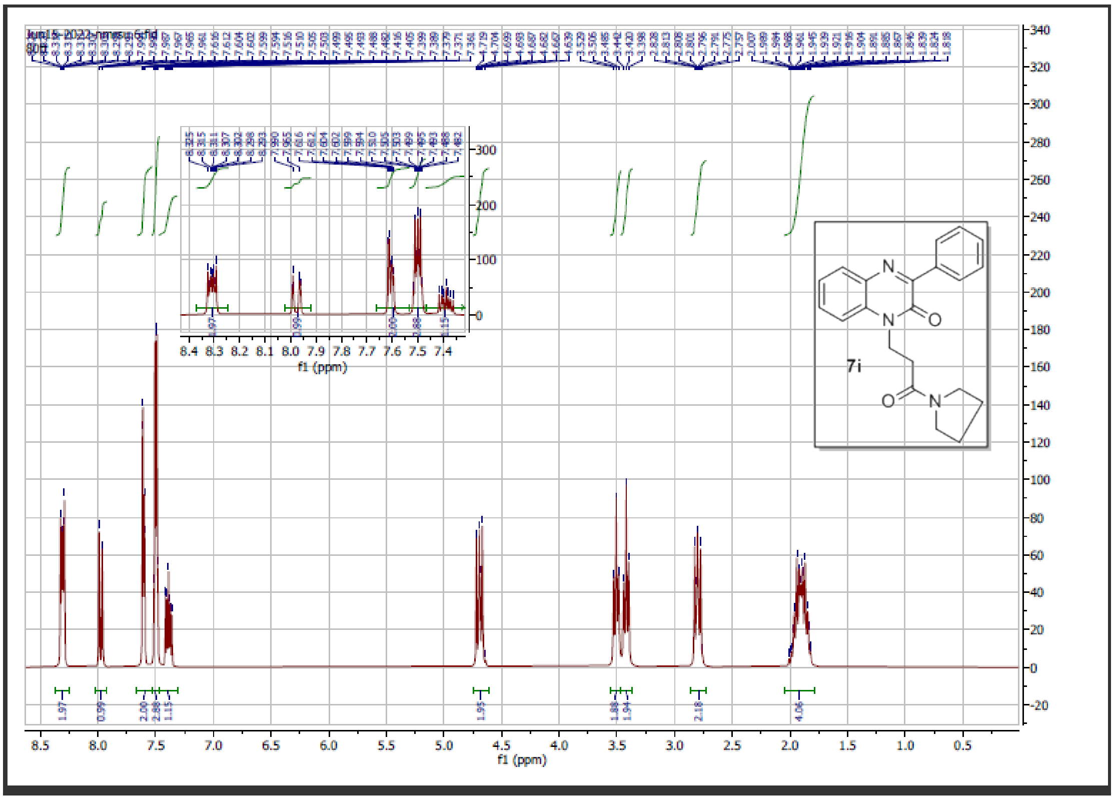

S35 ^{1}HNMR spectra of 7i

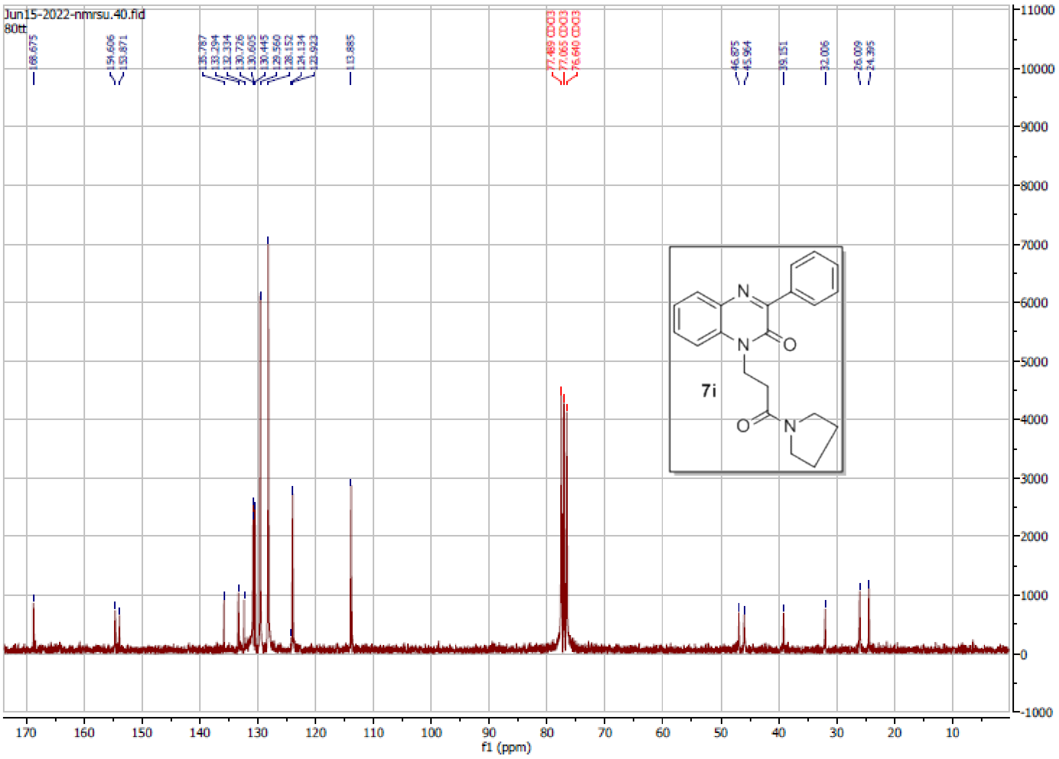

S36 ^{13}CNMR spectra of 7i

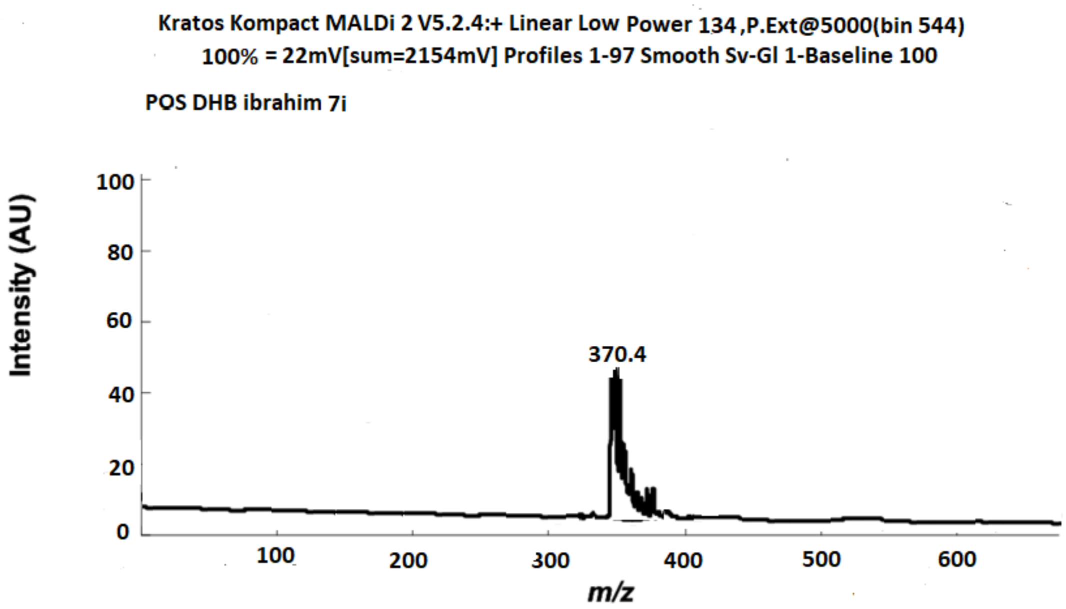

S37 MALDI spectra of 7i

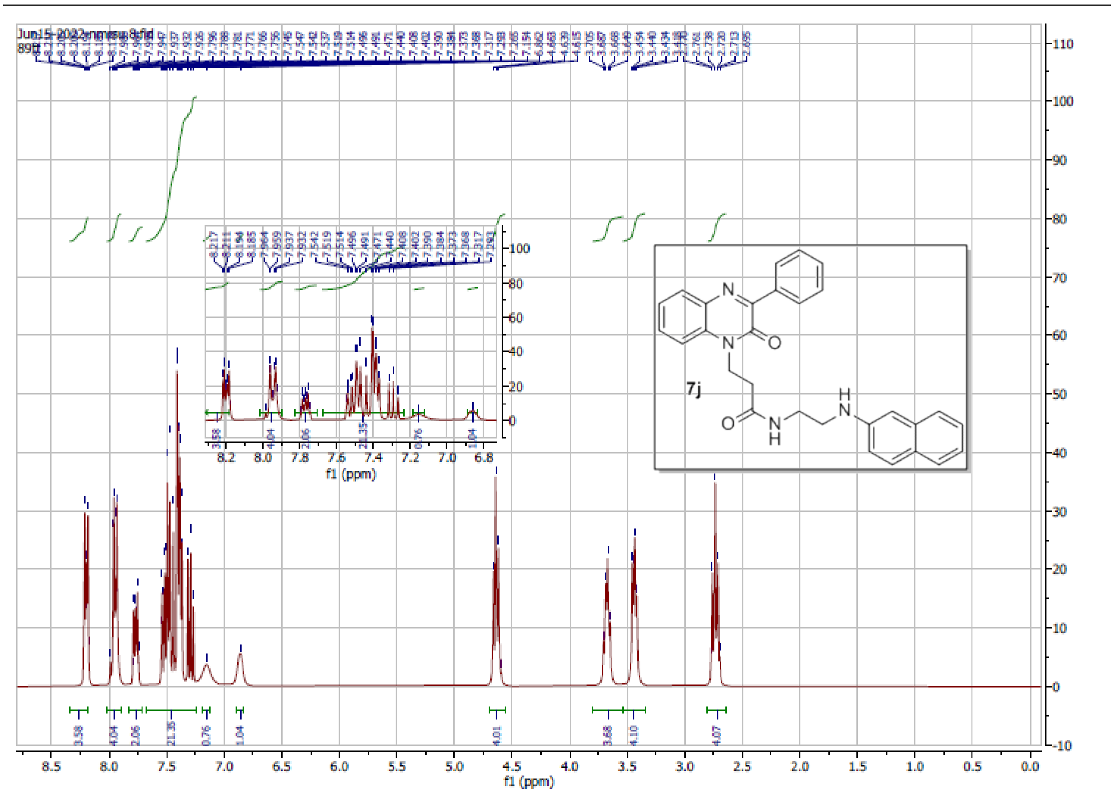

S38 ^{1}HNMR spectra of 7j

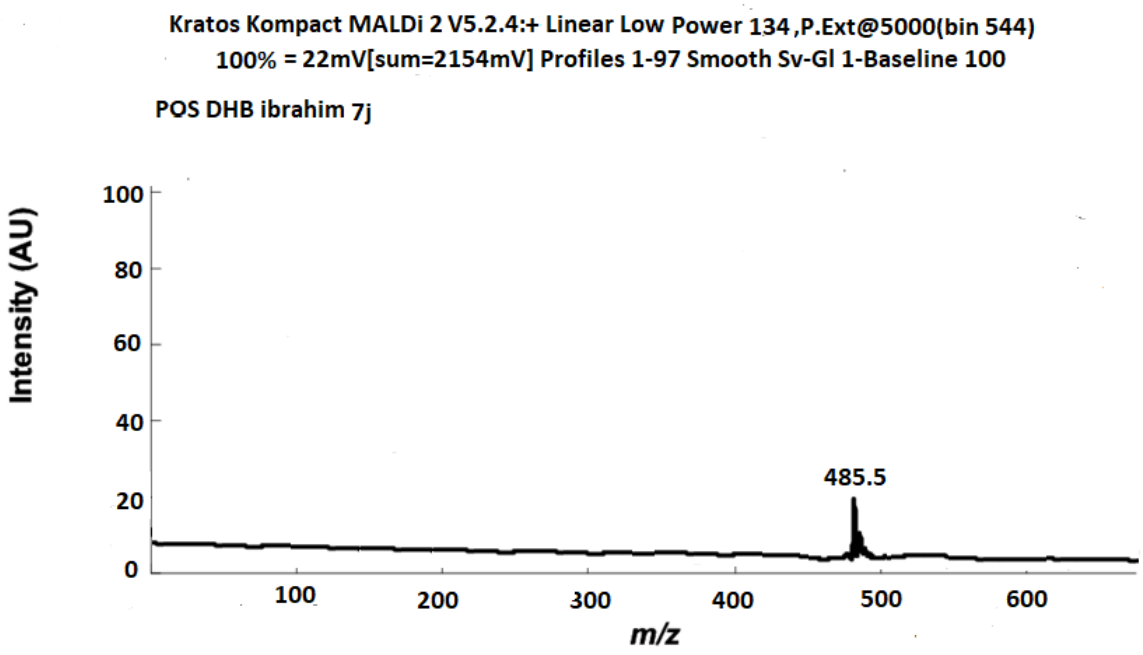

S39 MALDI spectra of 7j
